# Combining Niche and Dispersal in a Simple Model (NDM) of Species Distribution

**DOI:** 10.1371/journal.pone.0079948

**Published:** 2013-11-12

**Authors:** Michel Génard, Françoise Lescourret

**Affiliations:** INRA, UR1115 PSH, Avignon, France; University of Waikato (National Institute of Water and Atmospheric Research), New Zealand

## Abstract

Predicting the distribution of species has become a crucial issue in biodiversity research. Two kinds of model address this question: niche models, which are usually based on static approaches linking species distribution to habitat characteristics, and dispersal models, which are usually dynamic and process-based. We propose a model (NDM: niche and dispersal model) that considers the local presence of a species to result from a dynamic balance between extinction (based on the niche concept) and immigration (based on the dispersal concept), at a given moment in time, in a spatially explicit context. We show that NDM correctly predicts observed bird species and community distributions at different scales. NDM helps to reconcile the contrasting paradigms of metacommunity theory. It shows that sorting and mass effects are the factors determining bird species distribution. One of the most interesting features of NDM is its ability to predict well known properties of communities, such as decreasing species richness with decreasing patch size and increasing distance to the mainland, and the mid-domain effect at the regional scale, contrasting with predictions of much smaller effects at the local scale. NDM shows that habitat destruction in the matrix around patches of forest can affect the forest bird community, principally by decreasing the occurrence of typical matrix birds within the forest. This model could be used as the starting point for applied ecological studies on the management of species and community distributions.

## Introduction

Biodiversity is highly dependent on species distributions. The last 20 years have thus been marked by considerable interest in species distribution models (SDMs). SDMs have been used to predict the effects of habitat and landscape management and of climate change on species distribution [1], [2], [3]. Most SDMs are correlative models relating species occurrence to environmental predictor variables on the basis of statistically or theoretically derived response surfaces [2]. One of their most striking characteristics is their dependence on the niche concept. A frequent simplification in the field of SDMs involves considering SDMs to provide a *de facto* quantification of Hutchinson's realized niche for species, because the observed distributions are already constrained by biotic interactions and limiting resources. However, the metapopulation assumption that populations persist through subpopulation colonisation and extinction [4] implies that species distributions are temporally and spatially variable, even when habitat distributions are constant. It is therefore necessary to analyse species distribution as a function of dynamic processes, including dispersal as a key force, as proposed 50 years ago by MacArthur and Wilson [5] in their theory of dynamic equilibrium. Despite the status of dispersal as a key ecological constraint, this factor has rarely been incorporated into SDMs, and even then, generally only with a static approach [6].

By contrast, dispersal and related factors are commonly taken into account in metacommunity ecology [7]. Leibold *et al*. [8] have reviewed four approaches that have been put forward for understanding the dynamics of metacommunities, defined as sets of communities exchanging colonists of multiple species. The neutral approach assumes the per capita ecological equivalence of individuals, including their ability to disperse [9]. An interesting property of this approach is the mid-domain effect: a peak of species richness towards the centre of a region (a domain) accounted for by stochasticity and geometric constraints (boundaries) alone [10]. According to the patch dynamics approach, the environment is homogeneous and the coexistence of species is maintained by a trade-off between competitive ability and dispersal [11]. The species-sorting and mass-effect approaches are based on species ecological niches. In the species-sorting approach, environmental heterogeneity is considered strong enough to promote niche segregation between species [8]. Interacting species are at equilibrium, with the best competitor occupying each ecological niche. According to the mass-effect perspective, poor competitors can escape from local competitive exclusion by mass immigration. Species may therefore be present in both sink and source habitats [12].

The combination of dynamic dispersal and static niche approaches represents a major advance in models of species distribution [13]. Such a combination would help to reconcile the contrasting paradigms of metacommunity theory, and has been advocated by Winegardner *et al*. [14] as an advance in the field of metacommunity ecology.

The explicit consideration of space in studies of populations and communities is currently recognised as a major advance in contemporary ecology. Space is an important factor, and global self-organised spatial patterns may emerge, with unexpected consequences for population dynamics [15]. Following on from earlier attempts [1], several models have been recently proposed for the prediction of plant spatial distribution and dispersal [16], particularly in the contexts of environmental change [17],[18] and the spread of invasive plants [19].

We present here a simple model (NDM, for niche and dispersal model) combining niche and dispersal concepts in a spatially explicit context. NDM is simpler than the models mentioned above, because it focuses on the presence-absence of species, making it suitable for use in the many situations in which abundance data are not available. It is also different in being based on metacommunity theory and its four approaches. In addition, it is novel in closely connecting the distributions of single species and communities, by considering a community to be a collection of interacting species. We assessed the ability of NDM to simulate species and community distributions at different scales. We used the examples of (i) two passerine bird species with contrasting distributions (*Turdus pilaris* and *Anthus pratensis*) at a regional scale (France), (ii) two partridge species (*Perdix perdix* and *Alectoris rufa*) at a local scale (Eastern Pyrenees), and (iii) a passerine community at a local scale (a valley of the Eastern Pyrenees). We then carried out simulation studies, to analyse the emergent properties of NDM. Making reference to the body of research initiated by MacArthur and Wilson [5], we first investigated mainland-island systems with various island sizes and distances to the mainland, and possible mid-domain effects (see above) in virtual bird communities. We then evaluated NDM, applying it to the local passerine community, to assess the effect of matrix (non-forest) destruction on the composition of a forest bird assemblage.

## Materials and Methods

### The niche and dispersal model (NDM) of species distribution

NDM focuses on the presence or absence of species, by considering the behaviour of a typical individual within a particular species. The term “species” is used to denote this typical individual. NDM is based on an explicit representation of space through the use of a grid. As in Levins' model [20], the presence of a species in a given cell of the grid is the result of a dynamic balance between extinction and immigration at a given point in time. The principal characteristic of this system is that a species cannot persist in a given cell in the absence of immigration, unless its extinction rate is zero. Moreover, as in the mass-effect approach, dispersal allows species to colonise cells in which they are poor competitors by movement from cells in which they are better competitors.

NDM assumes that, at a given instant in time, each occupied cell sends species to a set of neighbouring cells, and that extinction and immigration (*I*) are stochastic processes. NDM assumes that the extinction or its complement, the persistence (*PE*) of a given species in a cell already occupied or successfully colonised depends exclusively on the correspondence between its niche and environment characteristics, according to the species sorting approach (see above). There is no general formulation of this dependence, which has indeed often been empirically established. Moreover, the environmental characteristics to be taken into account depend on scale and, consequently, on cell size. At a large regional scale, the cells are usually very large and we can assume that species persistence is affected only by biogeographic factors, such as mean temperature [21]. At a local scale, microhabitat descriptors, such as vegetation structure, play a prominent role. Depending on the number of variables involved, the expression of this probability of persistence may be very simple (e.g., 0 below the threshold for a single explanatory variable and 1 otherwise) or include a diverse range of habitat descriptors handled by GLM techniques, which have been shown to be powerful statistical methods for predicting the suitability of bird habitats [6].

The probability 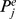 that cell *e* is successfully colonised by a species *j* emigrating from *n* neighbouring cells, the resultant of immigration and extinction forces, as stated above, can be represented by the product of 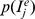, the probability that species *j* immigrates to cell *e* from *n* neighbouring cells, and 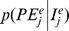
the conditional probability that *j* persists in *e* after immigration:



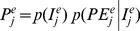
(1)


Assuming that immigration and persistence are two independent events, then 
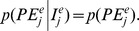


The *n* neighbouring cells are those at a distance from *e* of up to 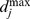, the maximal dispersal distance of species *j*, defined as a number of cells. If we consider rectangular cells, for example, each cell has eight neighbours for 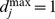
cell, 24 neighbours for 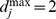 cells, etc. More generally, the number of neighbouring cells in a square of side 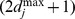 minus one cell (the central cell), is 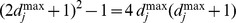
cells.

According to probability theory, when we consider two independent events, A and B, the probability that A or B occurs is:
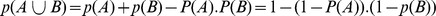



Using this rule of probability, 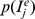, the probability that species *j* immigrates to cell *e* from *n* neighbouring cells, can be calculated from 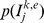, the probability that species *j* immigrates to cell *e* from cell *k*, with k varying from 1 to n:



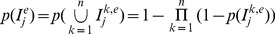
(2)


**Figure 1:**
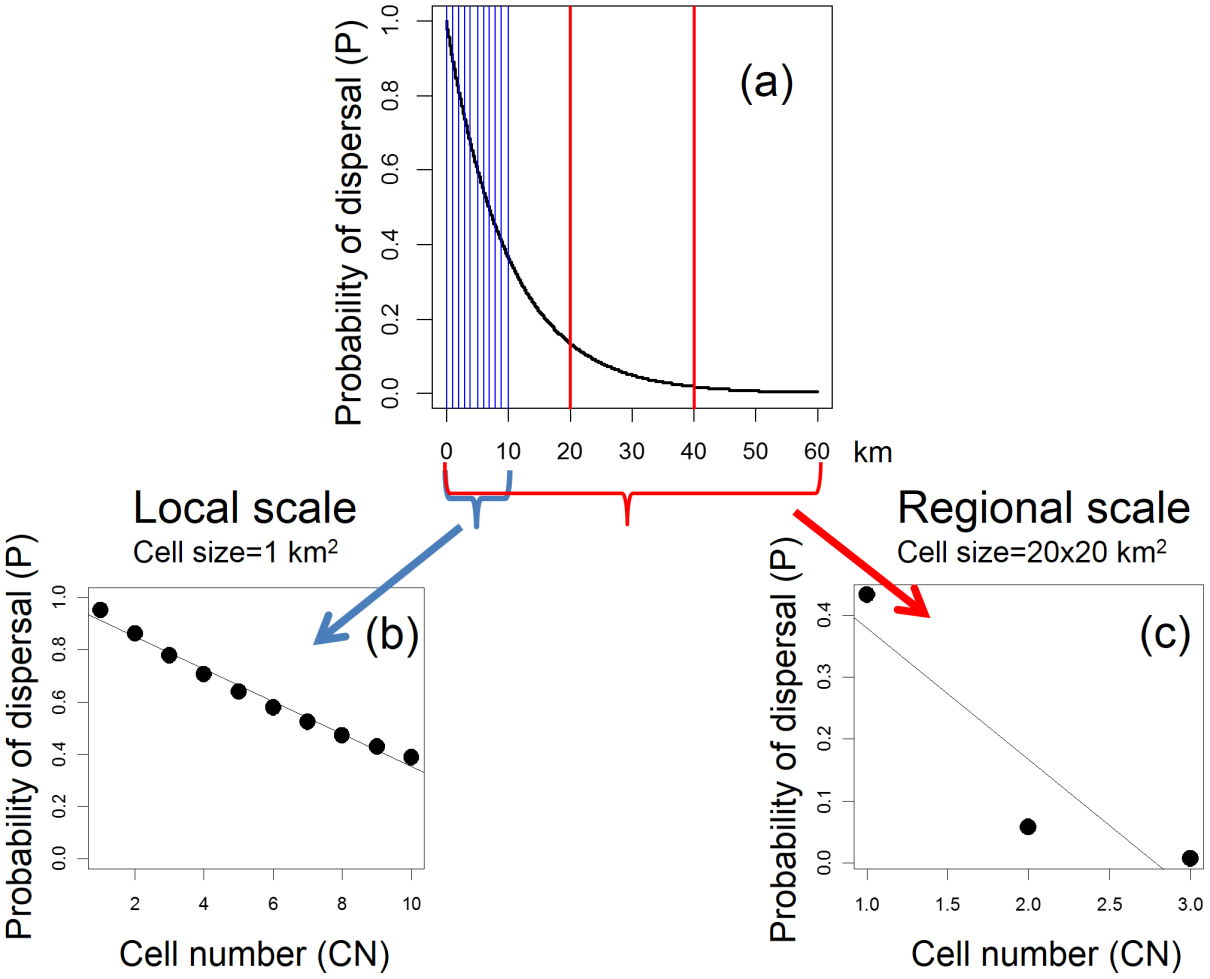
Dispersal at local and regional scale. (A) Typical probability of dispersal adapted from Sutherland *et al*. [23]. Dashed blue lines discretise the probability of dispersal, every 1 km, and red lines every 20 km. This discretisation is used to calculate the mean probability of dispersal as a function of the distance expressed in number of cells from the source. Discrete representation of the variation of the mean probability of dispersal at local (B) and regional (C) scales.


The probability of dispersal has been the subject of many theoretical and field studies (see Clobert *et al*. [22] for an overview). The negative exponential function is frequently used to describe the probability of dispersal. For instance, it has been used to model the dispersal of birds and mammals via a Poisson process [23]. Using avian examples, we show, in figure 1, that the negative exponential function can be approximated by a linear function at the local or regional scale. We therefore assume that the probability of the immigration of species *j* to cell *e* from cell *k* is maximal (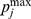) when the distance from cell *k* to cell *e* (*d_k,e_*), defined as a number of cells, is equal to 1, decreasing linearly thereafter with this distance:

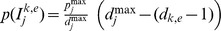
 if 1 ≤ *d_k,e_* ≤ 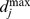




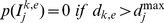
(3)


Equations (1–3) are valid for a community seen as a collection of non-competing species. However, interspecific competition plays a key role in shaping communities and limits the number of species that can fit into a particular community before the niche space is saturated [24]. Interspecific competition is taken into account further through the notion that there is a maximal species richness associated with each of the main habitat classes in a study area. Species richness is known to depend on habitat diversity [24] and we can assume, according to the principle of competitive exclusion [24], that when saturation (maximal richness) is reached at a particular site, new species cannot become established, even if the environment is suitable. In NDM, when several species occupy a cell, they are sorted according to their probability of persistence and the species are allowed to remain in the cells, according to this sorting, provided that maximal species richness has not yet been reached. As a consequence, the true probability that cell *e* is occupied by species *j* is less than 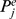
and species already present can be excluded.

Maximal dispersal distance (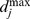) may be species-specific or can be calculated assuming either neutral or patch dynamics. In the neutral theory, the hypothesis of functional equivalence implies demographic identity on a per capita basis, in terms of dispersal in particular [9]. This led Chisholm and Lichstein [25] to assume that all individuals have the same dispersal kernel. The application of neutral principles to dispersal in the case dealt with by NDM, which focuses on species rather than individuals, assumes that all the species have the same dispersal ability (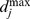 is constant, regardless of species). Baiser *et al*. [26] used the same principle in their neutral model of multitrophic metacommunity. As explained above, in the patch dynamics perspective, the coexistence of species is assumed to be maintained by a trade-off between competitive ability and dispersal [11]. This is taken into account in NDM, by assuming that 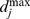
is inversely proportional to *C_j_*, the competitive ability of species *j* ranging from 0 (low competitive ability) to 1 (maximal competitive ability):



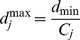
(4)where *d_min_* is the dispersal distance for a competitive ability of 1.

As explained above, the most competitive species in NDM are those with the highest probability of persistence. Accordingly, competitive ability, *C_j_,* is approximated by the maximal probability of the persistence of species *j* estimated for the study area.

The algorithm used to apply the equations and principles described above is a discrete-time algorithm, summarised as follows. We begin with an initial state for each cell of the grid: the presence-absence of each species. Then, for each instant *t* of a given period and each species and each cell, the probability of a species immigrating to the cell (if not already present) and then its probability of persistence in the cell are calculated. The probability of occurrence in the cell is the product of these two probabilities if the species is not already present (Eqn 1) and the probability of persistence if it is already present. The presence or absence of the species in the cell at *t+1* is defined by randomly drawing from a binomial distribution based on this probability of occurrence (Bernoulli trial). When there are several species in the set studied, the species are allowed to colonise (or remain in) a cell as a function of the sorting of their probabilities of persistence in the cell concerned, provided that maximal species richness has not yet been reached. The output of this step is the distribution of occurrences of the set of species in each cell of the grid at *t+1*, which serves as the starting point for the next cycle.

### Species and habitat distribution

We used NDM to analyse species distribution at a regional scale (France) and at two local scales (the Eastern Pyrenees as a whole and the Vanera valley (42° 23′ N, 2° E), a small valley in the Eastern Pyrenees).

At the scale of France, we studied changes in the distribution of breeding *Turdus pilaris* and *Anthus pratensis* from 1973 to 1988, using the occurrence data presented in the Atlas of Breeding Birds of France of Yeatman [27] and Yeatman-Berthelot and Jarry [28]. France was represented by 1056 grid cells of 540 km^2^ each. *Turdus pilaris* was chosen for study because of its recent strong expansion in France, and its continuous distribution, whereas *Anthus pratensis* was chosen as an example of a species with limited expansion and a patchy distribution. France was divided into two zones according to a threshold of 20°C for mean July temperature. This division made it possible to represent Mediterranean and south-western areas correctly, as*Turdus pilaris*, a species limited to subarctic, boreal and fresh temperate zones [29], cannot breed in these areas.

At the scale of French Eastern Pyrenees, we investigated the distributions of partridges (*Perdix perdix* and *Alectoris rufa*), which are rare in the Pyrenees and even declining in number (*P. Perdix*), over an area of 1068 km^2^. We made use of the precise partridge occurrence data published by Génard and Lescourret [30] (dataset S1). This area had an altitude of 1000 to 2900 m and was represented by 267 grid cells of 4 km^2^ each. Data for the orientation, topography, slope and coverage with seven principal vegetation types (alpine grassland, subalpine grassland, subalpine heath, montane grassland, montane heath, lowland forest and lowland open habitats) were available for each cell [30] (dataset S1).

At the scale of the Vanera valley, we studied the distribution of breeding bird communities, using bird census data (dataset S2) collected from 5350 ha of heterogeneous land at altitudes of 1100 to 2600 m [31]. Birds were sampled in 1982 by the point-count technique [32]. We recorded singing passerines and Picidae during a 20-minute period in the spring, and 214 point-counts were carried out in the centre of a 0.25 km^2^ cell. The cells were on a grid covering the study area (dataset S3). Habitat descriptors were evaluated within a radius of 50 m around the site of the point-count (dataset S2). Percentage herbaceous plant cover and ligneous plant cover to heights of 0–1 m, 1–4 m and more than 8 m were estimated by comparison with reference drawings representing imaginary cover levels of 5%, 10% and so on [33]. The altitude and the presence-absence of pine forest were also recorded. Agglomerative hierarchical clustering of the habitat dataset was performed and five habitat types were defined: lowlands (open fields, bocage and deciduous forest patches), heath, grassland, dense pine forest and clear pine forest (*Pinus uncinata* Ram and *Pinus sylvestris* L). Pine forest covered 30% of the study area. The maximal species richness per cell calculated for each habitat type was 17 for lowlands, 16 for heath, 11 for grasslands and 14 for dense or clear pine forests.

### Estimation of NDM parameters and of species persistence

For studies at the scale of France, we estimated, for each of the two species studied, the maximal probability of immigration, *p^max^*, and the maximal dispersal distance *d^max^*, which was assumed to be species-specific. For studies at the scales of the French Eastern Pyrenees and the Vanera valley, *p^max^* was fixed to one for all species, because the cells were small enough to assume that each of the species present occupied the entire area of the cell and, thus, had full potential to colonise the closest neighbouring cells. In the case of partridges at the scale of the French Eastern Pyrenees, we estimated species-specific *d^max^*. For the passerine community at the scale of the Vanera valley, we assumed that dispersal distance could be estimated by either a neutral or a patch dynamic approach. We therefore estimated a unique *d^max^* for the neutral model and a unique *d_min_* (dispersal distance for maximal competitive ability) for the patch dynamic perspective. Values of NDM dispersal parameters for the various cases studied are summarised in Table 1. Parameters *p^max^*, *d^max^* and *d_min_* were estimated by using the widely used pseudolikelihood approach introduced by Besag [34] 40 years ago to minimise a pseudomaximum likelihood fitting criterion relating observed bird occurrence and the simulated probability of occurrence of each species in each cell. This probability was calculated as the mean of 100 (bird communities) or 500 (other cases) simulations.

**Table 1:** Values of NDM dispersal parameters. Parameters were either fixed (fi) or estimated by minimising a fitting criterion (est).

Scale - Study area	Bird species	*p^max^*	*d^max^*
Regional - France	*Turdus pilaris*	0.0175 (est)	3 cells or 82 km (est)
	*Anthus pratensis*	0.011 (est)	2 cells or 55 km (est)
Local – French Eastern Pyrenees	*Perdix perdix*	1 (fi)	1 cell or 2.4 km (est)
	*Alectoris rufa*	1 (fi)	2 cells or 4.6 km (est)
Local – Vanera valley	All species	1 (fi)	7 cells or 4 km (est)

At the scale of France, we assumed that only biogeographic factors affected species persistence (see NDM description). Most of the cells occupied by *Turdus pilaris* and *Anthus pratensis* in the first survey (1973) were still occupied by these species (100 and 99%, respectively) in the second survey (1988). We therefore assumed that the probability of persistence of these species was 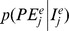 ≈1, except for *Turdus pilaris* in the Mediterranean area (cells with a mean July temperature of more than 20°C), for which the probability of persistence was assumed to be 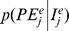  = 0.

For studies at a local scale (French Eastern Pyrenees and Vanera valley), the probability of persistence of bird species was estimated by linking habitat descriptors and species occurrence, through a stepwise generalised linear model (GLM) with binomial variance and logit link functions (Tables S1 and S2).

### Model simulations

NDM was implemented in S language, in R software [35]. In the case of species expanding their range in France, the model was run with a time step of one year, starting from the distribution observed in 1973. The distribution of partridges and that of the breeding bird community of the Vanera valley were assumed to be stable. Starting from the presence of each species in every cell, the model was run 100-500 times, to calculate the probability of occurrence of each species in each cell of the study area, approximated by averaging occurrences. The duration of simulations (50–100 years) was adjusted to reach stable distributions.

The structure of the simulated breeding bird community in the Vanera valley was studied by correlation network analysis. Each species was characterised by a vector expressing its probability of occurrence in the cells, as predicted by the model. A linear correlation coefficient was then used to characterise the link between vectors. The network consisted of a set of nodes (bird species) connected by a system of edges corresponding to the correlations (positive or negative) between species in cases in which the absolute values of the coefficient obtained exceeded 0.5 (α<0.001). The Kamada-Kawai algorithm was used for automatic layout generation [36]. The network is represented as a physical system, and the energy of the system was minimised by moving the nodes and changing the forces between them. The networks were constructed with Pajek graph drawing software [37], URL: http://vlado.fmf.uni-lj.si/pub/networks/pajek/.

Simulation studies of mainland-island systems and of mid-domain effects were performed at two different scales (cell areas of 1 and 400 km^2^) on a 30-species community occupying a homogeneous habitat. Starting from full occupancy of the habitat by all the species, NDM was run over a period of 50 years. The probability of persistence of each species was selected randomly, within the range 0.7–0.9, and maximum species richness per cell was fixed at 20. The probability of dispersal *p^max^* was assumed to be identical for all species, and to decrease from the local to the regional scale. Indeed, the home-range size of small passerine birds is 0.001–0.08 km^2^ [38], much smaller than the area of a cell at the regional scale. Thus, when only a small number of individuals of a given species are present, they occupy a small area of the cell, limiting the ability of the species concerned to disperse outside the cell. We therefore set the maximal dispersal probability *p^max^* to 1 at the local scale but to only 0.02 at the regional scale. The dispersal distance *d^max^* was selected at random from within a range of 1–6 cells (1–7 km at the local scale and 24–138 km at the regional scale).

NDM was used to analyse the effect of the landscape matrix on the composition of the Vanera forest bird community. The landscape matrix can take various forms and may contain habitats of various qualities [39]. Here, we use the term ‘matrix’ for non-forest habitat, in this case a mixture of open fields, bocage, heaths and grasslands. We assumed that the matrix could be destroyed (e.g. following a fire) thus becoming unfavourable to the birds (100% mortality). The model was run 100 times over a 50-year period, with either an intact or a destroyed matrix.

## Results

### NDM correctly predicts the spatial distribution of birds

**Figure 2:**
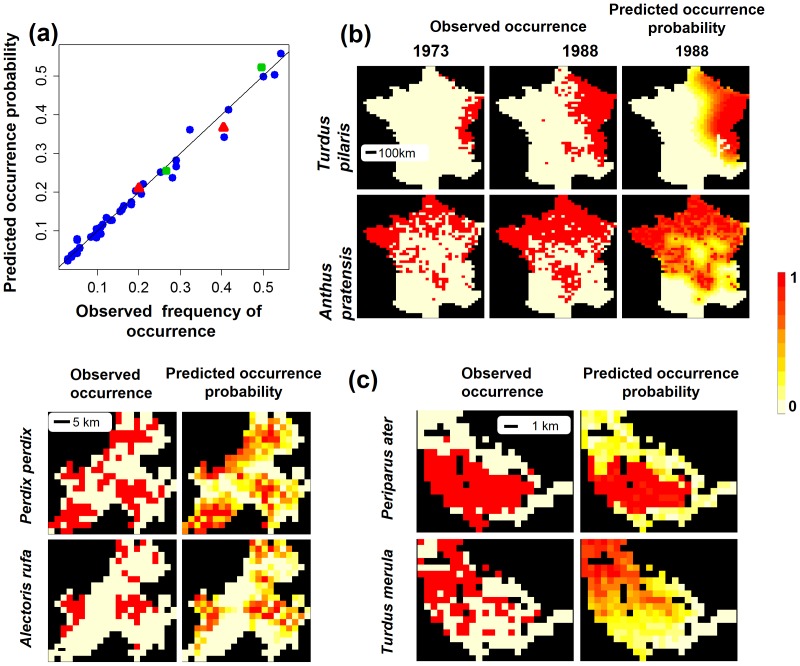
NDM prediction of species occurrence and spatial distribution at three scales. (a) Graph of predicted versus observed species occurrences (the 1:1 line is indicated). Green squares: *Turdus pilaris* and *Anthus pratensis* in France; red triangles: *Perdix perdix* and *Alectoris rufa* in the eastern Pyrenees; blue circles: breeding passerines in the Vanera valley. (b) Spatial distribution in France of *Turdus pilaris* and *Anthus pratensis* in 1988, either observed or predicted by NDM, using the observed 1973 distribution as the initial state. (c) Spatial distribution of *Perdix perdix* and *Alectoris rufa* in the eastern Pyrenees, and of *Periparus ater* and *Turdus merula* in the Vanera valley, either observed or predicted by NDM.

One of the key features of NDM is its ability to predict the percentage occurrence of a species regardless of scale (Figure 2a). At the scale of France, the expansion of the distributions of *Turdus pilaris* and *Anthus pratensis* from 1973 to 1988 was well predicted (Figure 2b). However, NDM failed to simulate the colonisation, by *Turdus pilaris*, of the mountain area in the south-central part of France, despite the occurrence of favourable July temperatures (below 20°C), probably because long-distance dispersal is not taken into account particularly well in equation 4. The maximal probability of immigration *p^max^* and the maximal dispersal distance 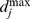 were estimated at 0.0175 and three cells (≈82 km), respectively, for *Turdus pilaris* and at 0.011 and two cells (≈55 km), respectively, for *Anthus pratensis* (Table 1). At the local scale, NDM correctly predicted the distribution of partridges in the Eastern Pyrenees and species distribution in the Vanera valley (Figure 2c). Maximum dispersal distance 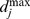 was estimated at one cell (≈2.4 km) for *Alectoris rufa* and two cells (≈4.6 km) for *Perdix perdix* (Table 1). *Perdix perdix* and *Alectoris rufa* exploited very different niches. *Perdix perdix* occupied non-forested habitats and corries facing south-west, south-east and west, whereas the habitat of *Alectoris rufa* was montane grassland and heath on south-west-facing moderate and steep slopes (Table S1).

We calculated dispersal distance for the species of the community of the Vanera valley from a patch dynamics perspective and from a neutral perspective. NDM gave similar, or even slightly better results for the neutral approach than for the patch dynamics approach (pseudomaximum likelihoods of 0.89 and 0.91, respectively). Dispersal distance was therefore assumed to be independent of the competitive ability of the species. The maximal distance of dispersal 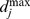 was estimated at seven cells for the bird species from the Vanera valley (≈4 km; Table 1).

### Insights into bird community structure

**Figure 3:**
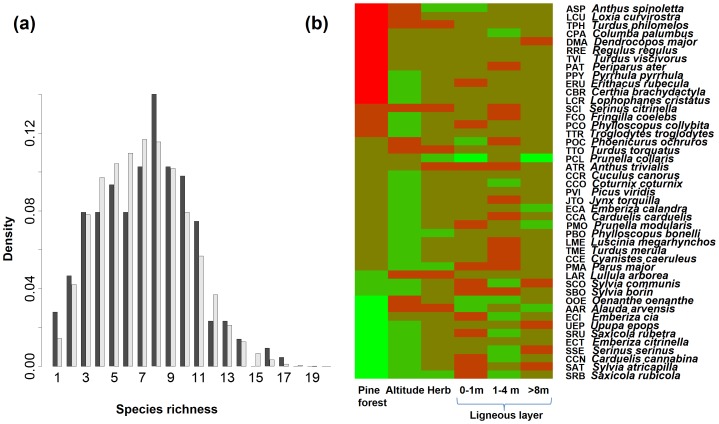
Vanera bird community structure. (a) Density of the probability of bird community richness per cell, either observed (black bars) or predicted by NDM (grey bars). The predicted distribution was generated from 500 simulations. (b) Niches of species in the bird community of the Vanera valley: heatmap of the coefficients selected by the stepwise GLM linking habitat descriptors to species occurrence. Each row represents a bird species and each column, a habitat descriptor. The label “Herb” indicates herbaceous plant cover. The values, which are standardised by the algorithm, increase from green to red.

The distribution of bird species richness in the Vanera valley predicted by NDM was not significantly different from the observed distribution (Chi^2^ test, *p*>0.1; Figure 3a). The birds of the Vanera valley explored very different niches, as shown by the habitat variables identified as significant in the stepwise generalised linear model (Figure 3b).

**Figure 4:**
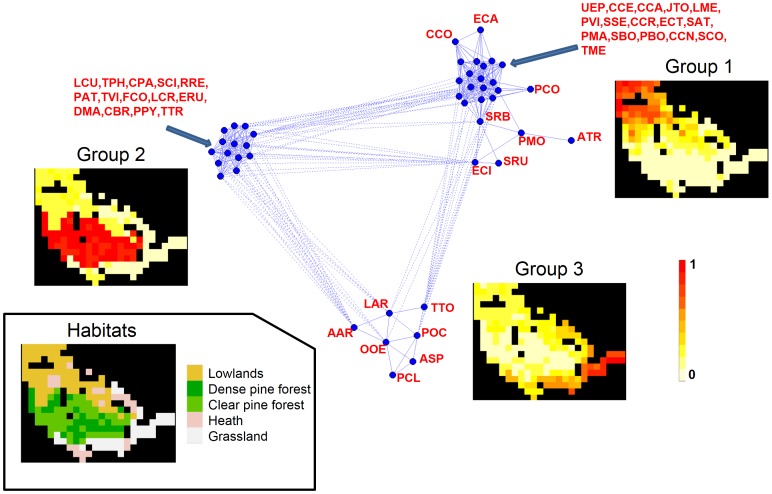
Correlation network for bird species predicted by NDM in the Vanera valley. The solid and dotted lines represent pairwise correlations with coefficients above 0.5 and below -0.5, respectively. The meaning of the species labels is indicated in figure 2b. The spatial distributions of the main habitats and groups of bird species defined on the basis of the correlation network analysis are shown. The colour indicates the mean probability of occurrence per cell for each group of species.

In the correlation network, 78% of the bird species were represented as highly connected nodes (more than 10 connections), highlighting the high degree of integration of this community. A clear pattern emerged, with three main groups of birds having highly correlated distribution patterns. These three groups had mutually exclusive spatial distributions (Figure 4). The first major group consisted of 24 species living in open fields, bocage, deciduous forest patches and heaths, such as *Cyanistes caeruleus, Sylvia atricapilla*, *Carduelis carduelis*, *Emberiza calandra* and *Prunella modularis*. Fourteen species living in pine forest (including *Periparus ater*, *Regulus regulus* and *Loxia curvirostra*) formed the second group, which was negatively correlated witheach of the other two groups. The third group contained seven species, including *Prunella collaris* and *Alauda arvensis*, present principally in open areas at high altitude.

### Effects of island size and distance to mainland and mid-domain effects are emergent properties of NDM

**Figure 5:**
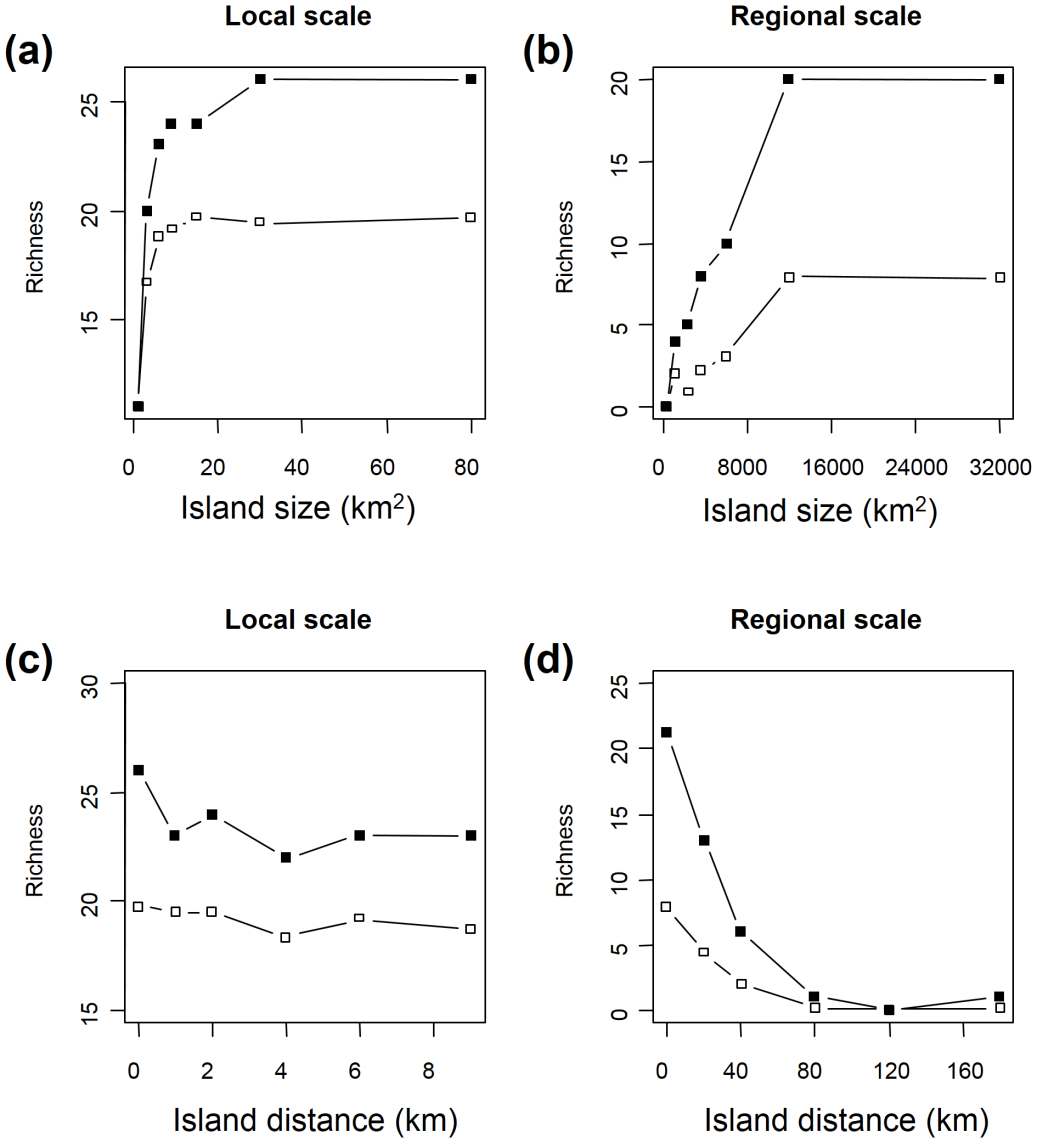
Species richness predicted by NDM depends on island size and distance to the mainland. Mean species richness per cell (open squares) and total species richness per island (closed squares) predicted by NDM 50 years after separation from the mainland. In (a) and (b), the islands were 4 and 80 km away from the mainland, at the local and regional scales, respectively. In (c) and (d), the island area was 6 km^2^ at local scale and 2400 km^2^ at regional scale.

When applied to a mainland-island system, one of the principal emergent properties of NDM was a clear decrease in species richness both per island cell and per island, with decreasing island size and increasing distance to the mainland, at the regional scale. By contrast, at the local scale, only very small islands (<3 km^2^) had a lower species richness than other islands, and isolation had only a slight effect (Figure 5).

**Figure 6:**
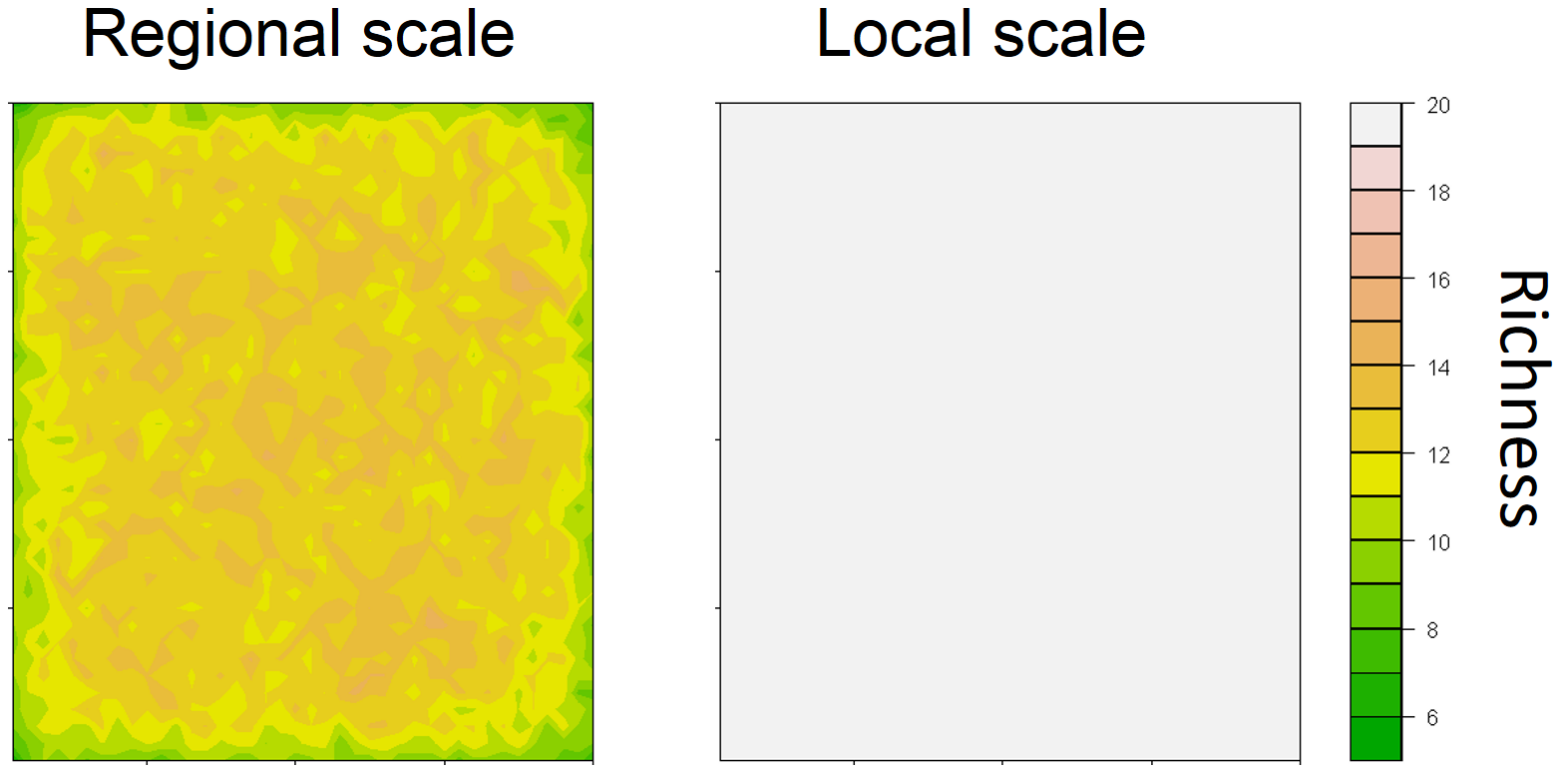
Simulation of spatial distribution of richness in a 30-species community occupying a homogeneous habitat. NDM predicts a mid-domain effect at the regional scale (characterised by a maximal probability of immigration – *p^max^* – of 0.02) and no mid-domain effect at the local scale (maximal probability of immigration – *p^max^* – of 1).

Considering the same community on a 40×40 grid corresponding to an area of 1600 km^2^ at the local scale and 640,000 km^2^ at the regional scale, simulations revealed that NDM generated a mid-domain effect at the regional scale, but not at the local scale (Figure 6).

### NDM predicts a contrasting effect of matrix destruction

**Figure 7:**
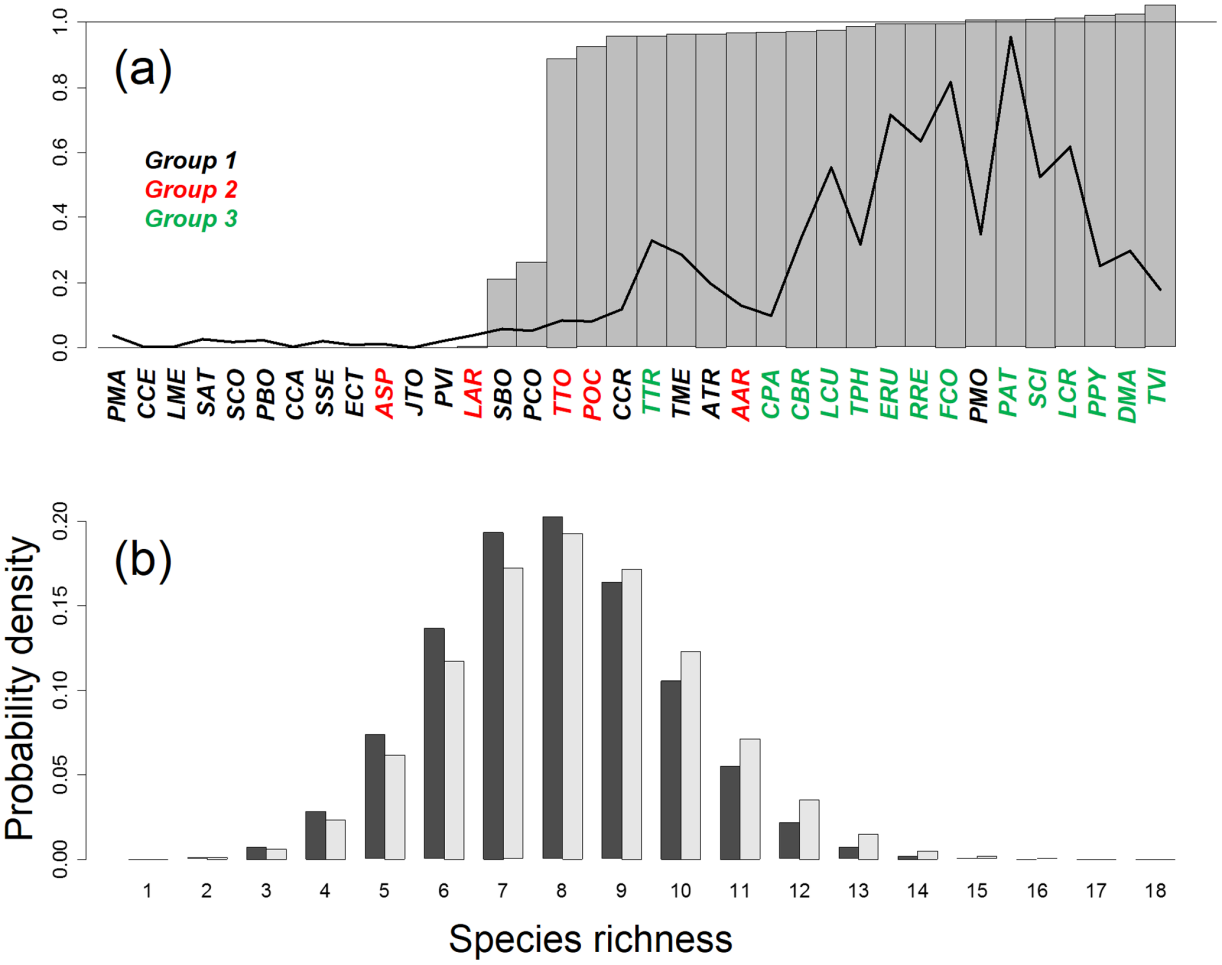
NDM prediction of the effect of the destruction of habitats surrounding the forest. (a) Mean probability of occurrence of each bird species in the Vanera valley forest before destruction (black line), and ratio of the mean probabilities of occurrence after and before destruction for each species (grey bars). The meaning of the species labels is indicated in figure 2b, with a colour code indicating the group to which each species belongs, as defined in figure 3. (b) Distribution of species richness per cell in the forest before (grey bars) and after (black bars) the destruction of habitats surrounding the forest. The simulated distributions result from 1000 simulations.

Following matrix destruction, total bird species richness in the forest decreased by 33% (from 36 to 24 species, Figure 7a). All the birds affected by matrix destruction were sparsely distributed in the forest (probability of occurrence less than 0.1). Eleven of the species that disappeared belonged to the bocage and deciduous forest group (group 1, Figure 4) and one species belonged to the high-altitude group (group 3, Figure 4). Two species of the bocage group (*Sylvia borin* and *Phylloscopus collybita*) and one species of the high-altitude group (*Lullula arborea*) persisted in the forest, but at a much lower probability of occurrence than before matrix destruction. Four species of the bocage and deciduous forest group (*Anthus trivialis*, *Cuculus canorus*, *Turdus merula*, *Prunella modularis*), three species from the high–altitude group (*Alauda arvensis*, *Turdus torquatus*, *Phoenicurus ochruros*) and all the species of the pine forest group persisted in the forest, with a probability of occurrence similar to that before matrix destruction. The number of species per forest cell 50 years after matrix destruction was, thus, only slightly lower than that before matrix destruction (7.8 vs. 8.2 species, on average, Figure 7b).

## Discussion

An understanding of species distribution pattern is a key element of community ecology [40]. The approach described here aims to integrate current knowledge into a dynamic view of spatial distribution patterns. It contrasts with most published SDMs, many of which are based on static statistical models. SDMs are based on the assumption that species distributions are in equilibrium with environmental conditions, but hybrid models combining dispersal features with traditional SDMs and referred to as dynamic range models by Schurr *et al*. [41], have proved to be superior to correlative models for the prediction of species spatial dynamics [42], [43]. Through the explicit consideration of space and dispersal and a restriction of the analysis to species presence/absence, we developed a model based on simple mathematical formalism and generated distribution maps for species and communities that closely matched the observed distributions. Of course, NDM has several limitations in its current form. For the sake of simplicity, we used a linear dispersal kernel. However, this choice precluded the precise consideration of long-distance dispersal (e.g. [44]) and was detrimental to the prediction of *Turdus pilaris* occurrence in one montainous region of central France. NDM could easily be modified to incorporate more realistic dispersal kernels. It could also be improved by better modelling of the niche. There is currently considerable discussion in this domain, concerning the need for functionally relevant predictors and the challenges remaining in terms of the modelling of presence-only data and model selection and evaluation [45]. Finally, the method use for the integration of interspecific competition and its effect on species assemblages is fairly basic and would benefit further refinement on the basis of the large amount of published data (e.g. [46]).

NDM was designed to describe interspecific interactions in metacommunities, which represents a major challenge for the future [41]. We think that NDM will help to bridge the gap between empirical and theoretical studies. Our NDM-based analysis showed that bird species distributions could be attributed to three of the four metacommunity paradigms, consistent with the review by Logue *et al*. [40] of observational and experimental approaches to metacommunities.

The application of NDM to a range of case studies showed clear niche segregation at the local scale, demonstrating the existence of a strong sorting effect. The occurrence of *Perdix perdix* reflected mild exposure, this species being found on deforested upland corries, whereas *Alectoris rufa* was found preferentially on montane grassland and heath on south-western-facing slopes, consistent with reported findings for these species in southern Europe mountains [47], [48]. The Vanera bird community also consisted of birds with contrasting niches. Regional niche variation may also occur [49], as shown for the bird communities of the Pyrenees and Alps [50]. Further investigations are required to account for regional niche variation in NDM, particularly as the available niche-based distribution models are not sufficiently accurate and spatially transferable [51]. For wide–ranging species, in particular, regional differences in ecological characteristics may cause apparent niche variation [49].

Contrasting with this major sorting effect, no significant trade-off was found between competitive ability and dispersal. There was probably no patch dynamics effect in our local case studies, due to high levels of environmental heterogeneity. As most of the habitats are suboptimal (probability of occurrence less than one), it is important to take into account a mass effect. The Vanera community would disappear if there was no dispersal. Along with sorting and mass effects, this community may display a neutral effect for dispersal distance. This is consistent with the similar magnitudes of the home ranges of the various passerine species of the Vanera community, given that there is a proportional relationship between median dispersal distance and territory size in birds [52]. The results obtained would be very different if the studied community included large birds of prey.

Another interesting finding was the ability of NDM to reproduce, at a regional scale, the effect of purely geometric constraints on the geography of species richness, leading to a mid-domain effect with no environmental gradient, as demonstrated theoretically by Colwell and Lees [53] and reported in several comprehensive studies on species distribution [54]. In addition, for mainland-island systems, NDM predicts a decrease in island species richness with increasing distance to the mainland and decreasing island area, as demonstrated in a number of studies [55]. This is also a key prediction of MacArthur and Wilson's Theory of Island Biogeography [5]. By contrast, we found no evidence of a mid-domain effect at the local scale, probably due to the high rate of dispersal at this scale, consistent with the predictions of Rangel and Diniz-Filho [10] in their theoretical study of neutral community dynamics. At the local scale, we observed a decrease in species richness only for very small patches, and no clear decrease in species richness with increasing distance to the main patch, suggesting that habitat may be the predominant factor at local scale. These findings are consistent with our previous results for bird communities in fragments of mountain forest [56]. Differences between the local and regional scales were related to maximal dispersal distance (*d^max^*) and maximal probability of immigration (*p^max^*). Maximal dispersal distances were estimated at about 2–5 km for the local scale and 50–80 km for the regional scale, consistent with published estimates for the dispersal of small birds [57], [52]. In NDM, *p^max^* is assumed to decrease with cell size, because the individuals of a species in a small cell (local scale) are likely to disperse from that cell with ease, whereas they are more likely to disperse within the cell when the cell is large (regional scale). Consequently, *d^max^* and *p^max^* may be considered important scaling factors in NDM.

It is now well established that local and regional community species richnesses are interdependent [58]. This interdependence will be taken into account in future studies on the NDM. Regional species richness is likely to affect local species richness in NDM, which focuses on the presence/absence of species, in that only the species present at regional scale can be present at local scale. Regions inevitably consist of a collection of localities, and thus, local processes must contribute to regional patterns. Logically, the regional *p^max^* should increase with the intensity of multisite dynamics for each species.

Recent studies have shown that the surrounding matrix has an important effect on biodiversity in fragmented habitats [59], particularly as concerns species richness in forest patches [60]. Consistent with these findings, NDM predicted a significant decrease in total Vanera forest species richness with increasing unsuitability of the matrix. Many matrix species can settle in the Vanera forest and maintain steady population levels, because the mass effect from the matrix compensates for high mortality rates in the forest. The degree to which the matrix differs from the forest alters the resource base and affects isolation differently for different species [39]. Consequently, when the matrix and the forest are very different, as in our case study, matrix destruction is likely to have only a weak impact on the abundance of true forest birds. Another important prediction of NDM is a possible contrasting effect of matrix destruction on total forest species richness and on (per cell) local forest species richness. This suggests that NDM could be used to resolve practical issues, such as the design of landscapes maximising biodiversity [61].

## Supporting Information

Table S1 Intercept and coefficients of the GLM (binomial variance and logit link) describing the probability of persistence of *Perdix perdix* and *Alectoris rufa* in the French Eastern Pyrenees according to habitat descriptors. The descriptors were coded in a semi-quantitative way (note between 0 and 9).
(DOC)

Table S2 Intercept and coefficients of the GLM (binomial variance and logit link) describing the probability of persistence of 45 species of the passerine community in the Vanera valley according to habitat descriptors.
(DOC)

Dataset S1 Presence-absence data of partridges (*Perdix perdix* and *Alectoris rufa*) and corresponding habitat variables in the French Eastern Pyrenees in 1985-1986.
(DOC)

Dataset S2 Presence-absence data of breeding bird communities and corresponding habitat variables in the Vanera valley (French Eastern Pyrenees, 42° 23′ N, 2° E) in 1982.
(DOC)

Dataset S3 Column (Nucol) and line (Nulig) coordinates to map 214 cells out of 302 from dataset S2 on a grid (19 lines and 24 columns). Code is to do the correspondence with dataset S2.
(DOC)
