# Supplementary material for: Combining Niche and Dispersal in a Simple Model (NDM) of Species Distribution
Source: PLoS One. 2013 Nov 12;8(11):e79948. doi: 10.1371/journal.pone.0079948 (PMC3827172; doi:10.1371/journal.pone.0079948)
Supplement: Table S1 — Intercept and coefficients of the GLM (binomial variance and logit link) describing the probability of persistence of Perdix perdix and Alectoris rufa in the French Eastern Pyrenees according to habitat descriptors. The descriptors were coded in a semi-quantitative way (note between 0 and 9). (DOC) [file pone.0079948.s001.doc]

**Table S1**. Intercept and coefficients of the GLM (binomial variance and logit link) describing the probability of persistence of *Perdix perdix* and *Alectoris rufa* in the French Eastern Pyrenees according to habitat descriptors. The descriptors were coded in a semi-quantitative way (note between 0 and 9).

|  | | *Perdix perdix* | *Alectoris rufa* |
| --- | --- | --- | --- |
| Intercept | | 0.3024 | -2.5409 |
| Exposure | S | -1.0251 | -0.2769 |
|  | S W | 0.3268 | 0.3032 |
|  | S E | 0.2140 | 0 |
|  | W | 0.3705 | 0 |
|  | N | 0 | -0.3082 |
|  | N W | 0 | -0.2487 |
|  | None | -0.2447 | 0 |
| Topography | Summit | 0.2423 | 0.3631 |
|  | Corrie | 3.1236 | -1.1012 |
|  | Valley bottom | 0.4401 | 0.4315 |
| Slope | Low | 0.1434 | 0 |
|  | Moderate | 0 | 0.3421 |
|  | Steep | 0 | 0.2323 |
| Vegetation | Forest | -0.3579 | -0.2853 |
|  | Lowland open habitat | -0.5418 | 0 |
|  | Montane heath | 0 | 0.1425 |
|  | Montane grassland | 0 | 0.6905 |
|  | Subalpine grassland | 0 | -1.0352 |
|  | Alpine grassland | 0 | -1.0047 |
