# Supplementary material for: Combining Niche and Dispersal in a Simple Model (NDM) of Species Distribution
Source: PLoS One. 2013 Nov 12;8(11):e79948. doi: 10.1371/journal.pone.0079948 (PMC3827172; doi:10.1371/journal.pone.0079948)
Supplement: Table S2 — Intercept and coefficients of the GLM (binomial variance and logit link) describing the probability of persistence of 45 species of the passerine community in the Vanera valley according to habitat descriptors. (DOC) [file pone.0079948.s002.doc]

**Table S2**. Intercept and coefficients of the GLM (binomial variance and logit link) describing the probability of persistence of 45 species of the passerine community in the Vanera valley according to habitat descriptors.

| Bird Species | Intercept | Altitude (m) | Pine  forest (boolean) | Herbaceous plant cover (%) | Ligneous plant cover 0-1m (%) | Ligneous plant cover 1-4m (%) | Ligneous plant cover > 8m (%) |
| --- | --- | --- | --- | --- | --- | --- | --- |
| *Parus major* | 11.9003 | -0.0089 | 0 | -0.0156 | 0.0185 | 0.0167 | 0 |
| *Cyanistes caeruleus* | 10.8214 | -0.0103 | 0 | 0 | 0 | 0.0296 | 0 |
| *Periparus ater* | 0.5776 | 0 | 2.1310 | 0 | 0 | 0.0193 | 0 |
| *Lophophanes cristatus* | 1.7754 | -0.0017 | 1.8341 | 0 | 0 | 0 | 0 |
| *Certhia brachydactyla* | 3.3533 | -0.0030 | 1.4022 | 0 | 0 | 0 | 0 |
| *Troglodytes troglodytes* | 2.1884 | -0.0021 | 0.7777 | 0 | 0 | 0 | 0 |
| *Turdus viscivorus* | -2.6719 | 0 | 1.1640 | 0 | 0 | 0 | 0 |
| *Turdus philomelos* | -5.3052 | 0.0012 | 1.0056 | 0.0180 | 0 | 0 | 0 |
| *Turdus merula* | 4.8953 | -0.0034 | 0 | 0 | 0 | 0.0128 | 0 |
| *Saxicola torquatus* | -5.2702 | -0.0030 | -8.7195 | -0.0217 | 0.0281 | 0.0267 | 0 |
| *Luscinia megarhynchos* | 24.2812 | -0.0196 | 0 | 0 | 0 | 0.0387 | 0 |
| *Erithacus rubecula* | 0.8916 | -0.0009 | 1.4261 | 0 | 0.0115 | 0 | 0 |
| *Sylvia atricapilla* | 6.2969 | -0.0063 | -1.4131 | 0 | 0.0174 | 0 | 0.0313 |
| *Sylvia borin* | 2.2574 | -0.0031 | -0.5124 | 0 | 0.0171 | 0.0140 | 0 |
| *Sylvia communis* | 7.3692 | -0.0074 | -0.7182 | 0 | 0.0527 | -0.0329 | 0.0189 |
| *Phylloscopus collybita* | 4.0150 | -0.0050 | 0.8369 | 0 | 0.0306 | 0 | 0 |
| *Phylloscopus bonelli* | 7.7894 | -0.0052 | 0 | -0.0335 | 0 | 0 | 0 |
| *Regulus regulus* | -1.6491 | 0 | 2.1867 | 0 | 0 | 0 | 0 |
| *Prunella modularis* | 0.4674 | -0.0007 | 0 | 0 | 0.0265 | 0 | -0.0131 |
| *Anthus trivialis* | -3.4740 | 0 | 0 | 0.0181 | 0.0121 | 0.0107 | 0 |
| *Carduelis carduelis* | 9.6181 | -0.0100 | 0 | 0 | 0 | 0.0412 | 0 |
| *Carduelis cannabina* | -4.8440 | -0.0044 | -8.8962 | 0 | 0.0252 | -0.0376 | 0 |
| *Serinus serinus* | 1.8264 | -0.0035 | -1.6665 | 0 | 0 | -0.0217 | 0.0396 |
| *Pyrrhula pyrrhula* | 1.5211 | -0.0020 | 1.0212 | 0 | 0 | 0 | 0 |
| *Fringilla coelebs* | 2.1840 | -0.0011 | 0.9802 | 0 | 0 | 0.0154 | 0 |
| *Emberiza citrinella* | -0.3944 | -0.0017 | -1.5370 | 0 | 0 | 0 | 0 |
| *Alauda arvensis* | -3.1627 | 0.0015 | -1.3939 | 0.0148 | -0.0142 | 0 | -0.0201 |
| *Turdus torquatus* | -11.1617 | 0.0032 | 0 | 0.0369 | 0 | 0 | 0 |
| *Saxicola rubetra* | -8.4866 | -0.0019 | -9.3483 | 0 | 0.0321 | -0.1156 | 0 |
| *Serinus citrinella* | -5.5404 | 0.0018 | 0.8930 | 0.0173 | 0 | 0.0109 | 0 |
| *Loxia curvirostra* | -8.6109 | 0.0035 | 2.4148 | 0 | 0 | 0 | 0 |
| *Emberiza cia* | -11.6846 | 0 | -9.5287 | 0 | 0.0356 | -0.0367 | 0 |
| *Phoenicurus ochruros* | -7.3847 | 0.0026 | 0 | 0 | -0.0208 | 0.0261 | 0 |
| *Lullula arborea* | -7.9531 | 0.0021 | -0.6579 | 0.0207 | 0 | 0 | 0 |
| *Anthus spinoletta* | -35.1209 | 0.0161 | 1.9438 | -0.0326 | -0.0380 | 0 | 0 |
| *Emberiza calandra* | 93.6412 | -0.0770 | 0 | 0 | 0 | 0 | -0.1234 |
| *Oenanthe oenanthe* | -12.9546 | 0.0015 | -9.6854 | 0 | -0.0191 | -0.1743 | 0 |
| *Prunella collaris* | 3.3138 | 0 | 0 | -0.1025 | -2.5277 | 0 | -2.7621 |
| *Dendrocopos major* | -3.4694 | 0 | 1.7254 | 0 | 0 | 0 | 0.0187 |
| *Jynx torquilla* | 12.0035 | -0.0122 | 0 | 0 | 0 | 0.0429 | 0 |
| *Picus viridis* | 7.8651 | -0.0068 | 0 | 0 | 0 | 0 | 0 |
| *Upupa epops* | 2.4229 | -0.0113 | -8.7075 | 0 | 0 | 0 | 0.0561 |
| *Coturnix coturnix* | 65.6529 | -0.0550 | 0 | 0 | 0 | -0.0555 | 0 |
| *Columba palumbus* | -2.8338 | 0 | 1.1234 | 0 | 0 | -0.0214 | 0 |
| *Cuculus canorus* | 8.4206 | -0.0059 | 0 | 0 | 0 | 0 | 0 |
