# Supplementary material for: Combining Niche and Dispersal in a Simple Model (NDM) of Species Distribution
Source: PLoS One. 2013 Nov 12;8(11):e79948. doi: 10.1371/journal.pone.0079948 (PMC3827172; doi:10.1371/journal.pone.0079948)
Supplement: Dataset S1 — Presence-absence data of partridges (Perdix perdix and Alectoris rufa) and corresponding habitat variables in the French Eastern Pyrenees in 1985-1986. (DOC) [file pone.0079948.s003.doc]

**Dataset S1**. Presence-absence data of partridges (*Perdix perdix* and *Alectoris rufa*) and corresponding habitat variables in the French Eastern Pyrenees in 1985-1986.

***Description of columns:***

*1) x, y=coordinates to map the data (only the first 231 data, for the other ones x and y get NA (not available).*

*2) P.perdix and A.rufa: 0-1 (absence-presence).*

*3) Then, 4 habitat variables splitted in modalities: distribution of 9 points (per cell) amongst the modalities of a variable (e.g. topography has 5 modalities and the sum of topography modalities per line is 9).*

- *From S to SE, plus AU=no exposure and CO=composite exposure: exposure.*
- *From VER to PLA: topography. VER=mountain side, SOM=summit, CIR=corrie, FON=valley bottom, PLA=flat area.*
- *From PFA to PFO: slope. PFA=low slope, PMY=moderate slope, PFO=steep slope.*
- *From FO to PAL: vegetation. FO=forest, OUV=lowland open habitat, LMO=montane heath, PMO=montane grassland, LSU=subalpine heath, PSU=subalpine grassland, PAL=alpine grassland.*

x y Pperdix Arufa S SW SE N NW NE WE ES AU CO VER SOM CIR FON PLA PFA PMY PFO FOR OUV LMO PMO LSU PSU PAL

1 1 0 0 0 0 2 5 0 0 0 0 2 0 7 0 0 0 2 2 2 5 0 0 0 0 0 0 9

1 2 1 0 0 0 0 5 0 4 0 0 0 0 9 0 0 0 0 0 7 2 0 0 0 0 5 0 4

3 1 1 0 0 0 0 0 4 1 0 0 4 0 5 0 0 4 0 4 5 0 0 0 0 0 8 0 1

2 2 1 0 0 3 5 0 0 1 0 0 0 0 9 0 0 0 0 0 1 8 0 0 2 0 4 0 3

4 1 1 0 1 3 1 0 0 0 2 0 2 0 7 1 0 1 0 2 2 5 0 0 0 0 5 4 0

3 2 0 0 0 0 1 0 0 0 0 8 0 0 9 0 0 0 0 0 0 9 4 0 5 0 0 0 0

2 3 1 0 0 0 1 2 1 4 0 0 1 0 5 1 0 3 0 1 2 6 6 0 0 0 1 0 2

4 2 1 0 0 2 6 0 2 0 0 0 0 0 7 1 0 1 0 2 1 6 1 0 2 0 4 2 0

3 3 1 0 0 4 0 0 0 0 5 0 0 0 5 1 0 3 0 0 5 4 6 0 0 0 3 0 0

5 2 1 0 2 4 2 0 0 0 0 0 1 0 5 1 1 2 0 1 4 4 0 0 0 0 4 3 2

4 3 1 0 0 0 0 9 0 0 0 0 0 0 9 0 0 0 0 0 0 9 5 0 0 0 1 0 3

3 4 1 0 2 6 0 0 0 0 0 0 1 0 6 2 0 1 0 2 2 5 5 0 0 0 2 2 0

6 2 1 0 0 0 1 0 5 0 0 0 3 0 6 3 0 0 0 5 3 1 1 0 0 0 3 0 5

5 3 1 0 1 2 5 0 0 1 0 0 0 0 7 0 1 1 0 0 4 5 7 0 0 0 2 0 0

4 4 1 0 0 5 4 0 0 0 0 0 0 0 7 0 0 2 0 6 3 0 2 0 0 0 7 0 0

3 5 0 1 3 1 5 0 0 0 0 0 0 0 6 0 0 3 0 7 1 1 0 4 5 0 0 0 0

7 2 1 0 2 0 0 0 0 0 0 3 4 0 3 4 1 1 0 4 1 4 0 0 0 0 3 0 6

6 3 0 0 0 0 0 1 0 0 2 0 6 0 3 0 0 0 6 6 1 2 3 0 0 0 4 0 2

5 4 1 0 0 1 6 0 0 0 0 0 2 0 5 2 1 1 0 4 5 0 1 0 0 0 0 1 7

4 5 1 0 0 1 5 0 0 0 0 0 3 0 6 1 0 0 2 5 3 0 2 0 0 0 0 7 0

3 6 0 1 1 2 1 0 0 0 0 0 5 0 4 0 0 5 0 6 3 0 0 9 0 0 0 0 0

7 3 0 0 1 0 0 1 0 0 0 0 7 0 3 3 0 0 3 7 2 0 0 0 0 0 0 2 7

6 4 1 0 0 0 4 0 0 0 0 3 2 0 5 2 1 1 0 2 4 3 0 0 0 0 0 3 6

5 5 1 0 0 0 0 0 0 6 0 3 0 0 6 2 0 1 0 6 3 0 4 0 0 0 0 5 0

4 6 1 1 0 0 8 0 0 0 0 0 1 0 6 0 0 2 1 4 5 0 0 6 3 0 0 0 0

8 3 0 0 4 0 0 0 1 2 0 1 1 0 5 1 1 2 0 1 4 4 0 0 0 0 1 3 5

7 4 1 0 0 0 5 0 0 0 0 4 0 0 5 0 0 4 0 6 1 2 0 0 0 0 0 9 0

6 5 1 0 0 0 5 0 0 1 0 3 0 0 8 0 0 1 0 4 5 0 0 0 0 0 5 4 0

5 6 0 0 3 3 3 0 0 0 0 0 0 0 6 3 0 0 0 1 2 6 0 3 2 0 4 0 0

4 7 0 0 0 0 0 0 0 0 0 0 9 0 0 0 0 0 9 9 0 0 0 9 0 0 0 0 0

8 4 1 0 0 1 3 0 0 0 0 0 5 0 2 4 0 3 0 6 3 0 1 0 6 0 0 2 0

7 5 1 0 0 2 0 0 0 0 0 0 7 0 2 0 0 0 7 7 0 2 0 0 0 0 1 8 0

6 6 0 0 0 0 0 0 1 0 0 0 8 0 1 0 0 0 8 8 0 1 0 0 0 0 0 9 0

5 7 0 1 2 0 1 0 1 0 0 0 5 0 2 2 0 0 5 5 2 2 0 2 4 3 0 0 0

4 8 0 0 0 0 0 0 0 0 0 0 9 0 0 0 0 3 6 9 0 0 0 9 0 0 0 0 0

3 9 0 0 0 0 0 0 0 0 0 0 9 0 0 0 0 3 6 9 0 0 0 9 0 0 0 0 0

2 10 0 0 0 2 0 4 3 0 0 0 0 0 7 2 0 0 0 0 9 0 9 0 0 0 0 0 0

9 4 0 0 0 0 3 0 0 0 0 0 6 0 3 0 0 0 6 6 3 0 1 0 0 0 1 7 0

8 5 0 0 0 1 0 0 1 0 5 0 2 0 6 1 0 0 2 5 4 0 5 0 0 0 1 0 0

7 6 0 0 0 0 0 0 3 2 0 0 4 0 5 0 0 2 2 4 5 0 2 0 0 0 3 4 0

6 7 0 0 2 0 3 0 0 0 0 0 4 0 5 0 0 3 1 4 5 0 0 6 3 0 0 0 0

5 8 1 1 0 0 0 0 0 0 0 0 9 0 0 0 0 9 0 9 0 0 0 9 0 0 0 0 0

4 9 0 1 0 0 0 0 0 0 0 0 9 0 0 0 0 4 5 9 0 0 0 5 2 2 0 0 0

3 10 1 1 0 0 0 0 2 6 0 0 1 0 6 1 0 2 0 1 6 2 4 0 5 0 0 0 0

9 5 0 0 0 1 5 0 0 0 0 1 2 0 7 0 0 0 2 2 4 3 7 0 0 0 2 0 0

8 6 0 0 0 4 0 0 0 0 0 0 5 0 4 0 0 4 1 5 4 0 9 0 0 0 0 0 0

7 7 0 0 0 0 0 0 0 0 0 0 9 0 0 0 0 2 7 9 0 0 4 0 0 0 4 1 0

6 8 1 1 1 1 0 1 0 1 0 0 5 0 4 0 0 2 3 5 4 0 0 0 8 1 0 0 0

5 9 0 1 0 0 0 0 0 0 0 0 9 0 0 0 0 5 4 9 0 0 0 9 0 0 0 0 0

4 10 1 1 0 0 2 0 0 3 2 0 2 0 5 3 0 1 0 3 5 1 5 0 0 0 4 0 0

3 11 0 0 0 0 0 1 1 7 0 0 0 0 4 0 0 5 0 0 8 1 0 9 0 0 0 0 0

10 5 0 0 0 1 1 0 0 0 0 2 5 0 4 4 0 1 0 5 0 4 6 0 0 0 3 0 0

9 6 0 0 0 0 2 0 0 6 0 1 0 0 7 2 0 0 0 1 8 0 6 0 0 0 1 2 0

8 7 0 0 0 4 1 0 0 0 0 0 4 0 4 1 0 3 1 8 1 0 9 0 0 0 0 0 0

7 8 0 0 0 0 0 0 0 0 0 0 9 0 0 0 0 2 7 9 0 0 0 9 0 0 0 0 0

6 9 1 1 0 0 0 0 0 0 0 0 9 0 0 0 0 5 4 9 0 0 0 5 2 2 0 0 0

5 10 1 1 0 4 0 0 2 0 2 0 1 0 7 1 0 1 0 2 6 1 1 0 0 2 5 1 0

4 11 1 1 0 3 0 0 0 3 0 0 3 0 6 3 0 0 0 3 0 6 1 0 0 0 8 0 0

15 1 0 0 0 2 2 0 0 3 0 0 2 0 6 0 0 1 2 2 7 0 4 5 0 0 0 0 0

14 2 1 0 0 0 1 2 0 0 0 2 4 0 3 2 4 0 0 6 1 2 1 6 0 2 0 0 0

10 6 0 0 0 0 0 0 0 0 0 0 9 0 0 0 0 0 9 9 0 0 5 3 0 0 1 0 0

9 7 0 0 0 0 0 0 0 0 0 0 9 0 0 0 0 0 9 9 0 0 9 0 0 0 0 0 0

8 8 0 0 0 0 0 0 0 0 0 0 9 0 0 0 0 3 6 9 0 0 9 0 0 0 0 0 0

7 9 0 0 0 0 0 0 0 0 0 0 9 0 0 0 0 0 9 9 0 0 0 5 1 3 0 0 0

6 10 1 1 0 2 0 1 5 0 0 0 1 0 6 0 0 3 0 1 3 5 1 0 2 0 4 2 0

7 11 1 1 0 0 0 0 2 3 1 0 3 0 4 3 0 2 0 3 5 1 4 0 0 0 2 3 0

4 12 1 0 0 3 1 0 0 2 0 0 3 0 5 3 0 1 0 3 5 1 0 0 0 0 3 1 5

15 2 0 0 0 1 1 0 1 5 1 0 0 0 9 0 0 0 0 0 5 4 8 0 0 1 0 0 0

11 6 0 0 0 0 0 0 0 0 0 0 9 0 0 0 0 0 9 9 0 0 1 8 0 0 0 0 0

10 7 0 0 0 0 0 0 0 1 0 0 8 0 1 0 0 0 8 8 1 0 2 3 0 0 0 0 0

9 8 1 0 0 0 0 0 0 0 0 0 9 0 0 0 0 0 9 9 0 0 8 1 0 0 0 0 0

8 9 0 0 0 0 0 0 0 0 0 0 9 0 0 0 0 4 5 9 0 0 0 9 0 0 0 0 0

7 10 0 0 0 0 0 0 8 1 0 0 0 0 9 0 0 0 0 2 6 1 9 0 0 0 0 0 0

6 11 1 1 0 6 0 0 2 0 1 0 0 0 6 0 0 3 0 0 2 7 2 0 0 0 5 0 2

16 2 0 0 0 0 2 4 1 0 0 0 1 1 4 2 0 3 0 1 0 8 9 0 0 0 0 0 0

15 3 1 1 0 0 0 0 1 1 2 0 5 0 4 0 0 4 1 9 0 0 0 5 4 0 0 0 0

14 4 0 0 0 0 0 0 0 0 0 2 7 0 2 0 0 1 6 7 2 0 5 0 4 0 0 0 0

13 5 0 0 0 0 2 0 3 0 0 0 4 0 5 0 0 0 4 6 2 1 8 0 0 1 0 0 0

12 6 0 0 0 0 0 0 3 0 0 0 6 0 3 0 0 6 0 6 3 0 1 8 0 0 0 0 0

11 7 0 0 0 0 0 0 4 2 0 0 3 0 5 0 0 1 3 3 6 0 6 3 0 0 0 0 0

10 8 0 0 0 0 1 0 2 3 0 0 4 1 1 3 0 2 3 9 0 0 9 0 0 0 0 0 0

9 9 1 0 0 0 1 0 0 0 3 0 5 0 4 0 0 2 3 9 0 0 0 7 1 1 0 0 0

8 10 0 0 0 0 0 3 4 0 2 0 0 0 8 0 0 1 0 0 9 0 8 1 0 0 0 0 0

7 11 0 0 0 3 0 0 0 2 0 4 0 0 5 4 0 0 0 4 2 3 0 0 0 0 4 0 5

16 3 0 0 2 0 0 3 2 0 0 0 2 0 7 0 0 0 2 2 2 5 6 0 3 0 0 0 0

15 4 1 0 0 0 0 0 6 2 0 0 1 0 3 4 0 1 1 6 1 2 5 0 4 0 0 0 0

14 5 1 0 0 2 0 0 6 0 0 0 1 0 5 2 0 1 1 3 5 1 9 0 0 0 0 0 0

13 6 1 0 0 0 0 0 4 0 0 0 5 0 4 0 0 0 5 5 3 1 9 0 0 0 0 0 0

12 7 1 0 0 2 2 0 0 2 0 0 3 0 6 2 0 1 0 4 2 2 4 0 0 0 5 0 0

11 8 0 0 0 0 4 0 0 0 0 3 2 0 7 2 0 0 0 8 0 1 6 2 0 0 1 0 0

10 9 1 0 0 0 0 8 1 0 0 0 0 0 8 0 0 1 0 9 0 0 8 0 0 0 1 0 0

9 10 0 0 1 0 2 0 1 1 0 0 4 0 4 1 0 4 0 4 0 5 7 1 1 0 0 0 0

8 11 1 0 0 1 0 0 2 3 3 0 0 0 8 1 0 0 0 1 0 8 4 0 0 0 2 2 1

17 3 0 0 3 0 0 5 0 1 0 0 0 0 9 0 0 0 0 0 1 8 9 0 0 0 0 0 0

16 4 0 0 0 0 0 0 0 1 0 0 8 0 0 0 0 6 3 8 1 0 3 6 0 0 0 0 0

15 5 1 0 0 0 0 4 3 2 0 0 0 0 3 4 0 2 0 8 1 0 3 0 6 0 0 0 0

14 6 1 0 0 0 0 0 8 0 0 1 0 0 6 0 1 2 0 0 5 4 2 0 0 0 7 0 0

13 7 1 0 0 4 0 0 2 0 0 0 3 0 5 0 0 1 3 3 3 3 7 0 0 0 1 1 0

12 8 1 1 0 4 2 0 0 1 0 0 2 0 7 0 0 2 0 2 0 7 4 3 2 0 0 0 0

11 9 0 0 0 0 0 0 5 3 0 0 1 0 8 0 0 1 0 1 5 3 1 3 1 2 2 0 0

10 10 1 0 0 2 7 0 0 0 0 0 0 0 2 2 3 2 0 0 0 9 6 3 0 0 0 0 0

9 11 1 0 0 1 0 0 0 3 4 0 0 1 8 1 0 0 0 0 0 9 4 1 0 0 3 1 0

8 12 0 0 0 0 0 0 8 1 0 0 0 0 8 0 0 1 0 0 1 8 1 0 0 0 3 3 2

17 4 0 0 0 2 1 0 0 4 0 0 2 0 7 0 0 0 2 2 1 6 8 1 0 0 0 0 0

16 5 0 0 0 0 0 0 5 2 0 0 1 1 5 2 0 2 0 2 7 0 7 0 2 0 0 0 0

15 6 0 0 0 0 1 1 4 2 0 0 1 0 8 0 0 1 0 1 2 6 8 0 1 0 0 0 0

14 7 1 0 0 0 3 0 0 0 0 2 4 0 2 0 2 1 4 6 2 1 1 0 1 0 3 1 3

13 8 1 0 0 5 1 0 0 1 0 2 0 0 7 1 0 1 0 0 4 5 3 0 0 0 4 2 0

12 9 1 1 0 6 0 0 0 0 2 0 1 0 6 0 0 3 0 1 4 4 2 7 0 0 0 0 0

11 10 1 0 0 0 0 2 1 4 0 0 2 0 5 3 0 1 0 2 5 2 5 0 3 0 1 0 0

10 11 0 0 0 0 0 5 4 0 0 0 0 0 4 0 0 5 0 0 3 6 7 0 2 0 0 0 0

9 12 1 0 0 0 2 1 4 1 0 0 1 0 8 0 0 0 1 1 1 7 0 0 0 0 2 1 6

8 13 0 0 0 0 0 0 2 1 0 0 6 0 3 0 0 4 2 6 0 3 0 0 0 0 2 1 6

19 3 0 0 0 1 0 0 1 1 0 1 5 0 4 0 0 4 1 5 1 3 2 7 0 0 0 0 0

18 4 0 0 0 4 0 0 1 1 1 0 2 0 7 0 0 1 1 2 0 7 8 1 0 0 0 0 0

17 5 0 0 0 1 0 0 1 2 3 0 2 0 7 0 0 2 0 2 4 3 5 4 0 0 0 0 0

16 6 0 0 1 0 1 2 3 1 0 0 1 0 7 0 0 2 0 1 5 3 7 2 0 0 0 0 0

15 7 1 1 0 0 0 2 0 4 0 3 0 0 7 0 0 2 0 3 6 0 4 0 0 0 5 0 0

14 8 1 1 0 2 2 0 0 3 0 0 2 0 5 1 0 3 0 3 4 2 2 0 5 1 1 0 0

13 9 1 1 0 3 5 0 0 0 0 1 0 0 7 0 0 2 0 0 3 6 0 2 4 0 3 0 0

12 10 0 1 2 3 2 1 0 1 0 0 1 0 4 1 0 4 0 1 2 6 5 3 0 1 0 0 0

11 11 0 0 1 1 0 0 6 0 0 0 1 0 8 0 0 1 0 1 2 6 6 3 0 0 0 0 0

10 12 0 0 0 0 0 1 1 7 0 0 0 0 6 1 0 2 0 1 0 8 7 0 0 0 2 0 0

9 13 0 0 0 0 0 0 6 3 0 0 0 0 6 2 0 1 0 0 6 3 1 0 0 0 5 1 2

19 4 0 0 0 0 0 0 5 3 0 0 1 0 8 0 0 0 1 1 1 7 9 0 0 0 0 0 0

18 5 0 0 0 2 0 0 2 1 3 0 1 0 6 0 0 2 1 2 2 5 5 4 0 0 0 0 0

17 6 1 0 0 0 0 0 3 3 2 0 1 0 5 3 0 1 0 1 3 5 6 0 3 0 0 0 0

16 7 1 0 0 0 4 0 0 5 0 0 0 0 9 0 0 0 0 1 7 0 6 0 1 2 0 0 0

15 8 1 1 0 3 5 0 0 0 0 1 0 0 6 0 0 3 0 1 3 5 0 0 5 0 4 0 0

14 9 0 0 0 3 0 0 0 4 0 0 2 0 7 2 0 0 0 3 3 3 9 0 0 0 0 0 0

13 10 0 1 0 2 1 0 0 0 5 0 1 0 6 1 0 2 0 1 6 2 3 6 0 0 0 0 0

12 11 0 1 0 0 2 0 3 2 0 0 1 0 7 1 0 1 0 1 5 3 4 5 0 0 0 0 0

11 12 0 0 0 1 0 0 4 4 0 0 0 0 9 0 0 0 0 0 1 8 4 0 2 0 2 1 0

10 13 1 0 0 1 2 0 5 1 0 0 0 0 7 1 0 1 0 0 6 3 3 0 0 0 3 0 3

19 5 0 0 0 0 4 2 0 0 0 0 3 0 6 0 0 0 3 3 0 6 6 1 2 0 0 0 0

18 6 1 0 0 1 2 0 0 2 0 4 0 0 5 3 0 1 0 0 3 6 6 0 3 0 0 0 0

17 7 1 0 0 4 2 0 1 0 0 1 1 0 8 0 0 1 0 4 4 1 3 0 4 2 0 0 0

16 8 1 0 1 0 0 2 2 2 0 0 2 0 6 0 1 0 2 2 5 2 4 0 3 0 0 2 0

15 9 1 1 0 6 0 0 0 1 0 0 2 0 7 0 0 1 1 2 0 7 4 1 4 0 0 0 0

14 10 0 1 0 2 4 0 0 1 0 0 2 0 3 1 0 3 2 2 5 2 6 0 3 0 0 0 0

13 11 0 0 2 0 2 2 2 0 0 0 1 0 8 0 0 1 0 1 1 7 3 6 0 0 0 0 0

12 12 0 0 0 3 0 0 3 3 0 0 0 0 9 0 0 0 0 0 0 9 6 1 2 0 0 0 0

11 13 0 0 0 1 2 2 0 4 0 0 0 0 7 0 0 2 0 0 2 7 4 0 0 0 1 4 0

10 14 1 0 0 1 0 0 3 5 0 0 0 0 4 2 0 3 0 1 6 2 0 0 0 0 4 4 1

19 6 0 0 0 0 4 0 0 1 0 1 3 0 5 1 0 1 2 3 4 2 5 1 3 0 0 0 0

18 7 0 0 0 0 0 2 7 0 0 0 0 0 8 0 0 1 0 0 4 5 8 0 1 0 0 0 0

17 8 0 0 5 0 1 0 0 2 0 0 1 0 8 0 0 1 0 1 5 3 3 3 2 1 0 0 0

16 9 1 1 0 5 2 0 0 0 0 0 2 0 5 1 0 3 0 2 7 0 1 8 0 0 0 0 0

15 10 0 1 0 0 0 2 3 4 0 0 0 0 8 0 0 1 0 0 1 8 9 0 0 0 0 0 0

14 11 0 1 3 3 3 0 0 0 0 0 0 0 6 3 0 0 0 0 3 6 3 6 0 0 0 0 0

13 12 0 0 0 0 0 0 0 0 0 1 8 0 0 0 0 2 7 8 1 0 3 6 0 0 0 0 0

12 13 1 0 0 0 0 0 9 0 0 0 0 0 5 0 0 4 0 0 2 7 7 0 2 0 0 0 0

11 14 0 0 0 0 0 4 3 0 0 0 2 0 3 3 0 3 0 2 4 3 4 0 0 0 1 4 0

19 7 1 0 0 0 0 0 1 4 2 0 2 0 6 0 0 2 1 2 3 4 3 1 4 1 0 0 0

18 8 0 0 1 0 2 0 0 1 0 2 3 0 6 1 0 0 2 4 2 3 0 0 8 1 0 0 0

17 9 0 0 0 0 3 2 0 4 0 0 0 0 9 0 0 0 0 0 0 9 5 2 2 0 0 0 0

16 10 0 1 2 3 3 0 0 0 0 0 1 0 4 1 0 3 1 2 4 3 0 8 1 0 0 0 0

15 11 0 1 0 2 1 0 0 0 0 0 6 0 3 0 0 0 6 6 2 1 3 4 2 0 0 0 0

14 12 0 0 0 0 0 0 2 1 2 0 4 0 4 4 0 1 0 4 5 0 3 6 0 0 0 0 0

13 13 0 0 0 0 0 0 4 5 0 0 0 0 7 1 0 1 0 0 3 6 5 0 4 0 0 0 0

12 14 0 0 0 0 1 4 2 2 0 0 0 0 7 0 0 2 0 1 4 4 3 0 5 0 1 0 0

11 15 1 0 0 0 1 1 0 2 0 1 4 0 1 0 0 4 4 4 5 0 1 0 0 0 0 5 3

20 7 0 0 0 0 1 0 5 3 0 0 0 0 9 0 0 0 0 6 3 0 9 0 0 0 0 0 0

19 8 1 0 0 0 0 0 1 0 0 0 8 0 1 0 0 4 4 9 0 0 4 2 3 0 0 0 0

18 9 0 1 1 3 3 0 0 0 0 0 2 0 2 3 0 1 2 8 1 0 1 8 0 0 0 0 0

17 10 0 1 0 0 0 0 0 6 0 3 0 0 9 0 0 0 0 0 8 1 1 3 5 0 0 0 0

16 11 0 0 1 2 0 2 0 2 0 0 2 0 6 0 0 3 0 2 0 7 6 2 1 0 0 0 0

15 12 0 0 0 2 1 0 1 0 0 1 4 0 5 0 0 3 1 4 2 3 3 6 0 0 0 0 0

14 13 0 0 0 0 2 0 0 3 0 0 4 0 5 1 0 3 0 9 0 0 5 4 0 0 0 0 0

13 14 0 0 0 1 4 0 1 0 0 1 2 0 7 0 0 2 0 2 0 7 0 7 2 0 0 0 0

12 15 0 0 0 0 4 1 1 3 0 0 0 0 7 0 0 2 0 0 3 6 6 0 2 0 1 0 0

11 16 1 0 0 0 4 1 0 3 0 0 1 0 7 1 0 1 0 2 2 5 0 0 0 0 3 6 0

20 8 0 0 1 5 3 0 0 0 0 0 0 0 9 0 0 0 0 0 9 0 3 6 0 0 0 0 0

19 9 0 0 0 0 0 0 2 0 0 0 7 0 2 0 0 2 5 7 2 0 0 0 9 0 0 0 0

15 13 0 0 0 0 0 0 1 0 0 0 8 0 1 0 0 8 0 8 1 0 4 5 0 0 0 0 0

14 14 1 1 0 4 2 0 2 0 0 0 0 1 5 3 0 1 0 1 3 5 0 5 4 0 0 0 0

13 15 0 1 1 4 0 1 3 0 0 0 0 0 8 0 0 1 0 0 3 6 4 0 3 1 1 0 0

12 16 1 0 0 0 2 2 3 0 0 0 2 0 6 0 0 1 2 2 5 2 0 0 0 0 2 7 0

11 17 1 0 0 0 5 2 0 2 0 0 0 0 8 0 0 1 0 1 4 4 7 0 2 0 0 0 0

21 8 0 0 0 0 0 0 5 4 0 0 0 0 9 0 0 0 0 5 4 0 8 1 0 0 0 0 0

19 10 0 0 0 1 2 0 1 2 0 0 3 0 6 0 0 0 3 9 0 0 0 9 0 0 0 0 0

15 14 0 1 0 3 0 2 3 0 0 0 1 0 5 0 0 4 0 1 4 4 4 5 0 0 0 0 0

14 15 1 0 0 1 0 0 1 7 0 0 0 0 6 1 0 2 0 1 5 3 8 0 0 0 1 0 0

13 16 1 0 0 0 1 0 4 2 0 0 2 0 6 0 0 1 2 2 6 1 4 0 0 0 2 3 0

12 17 1 1 0 1 1 0 0 5 1 0 0 1 0 1 0 0 0 2 7 0 6 0 2 0 1 0 0

21 9 0 0 1 3 3 0 0 0 0 0 3 0 6 0 0 0 3 3 6 0 4 5 0 0 0 0 0

20 10 0 0 0 0 0 0 3 2 0 2 2 0 7 0 0 1 1 4 5 0 0 9 0 0 0 0 0

19 11 0 0 0 7 2 0 0 0 0 0 0 0 9 0 0 0 0 6 3 0 2 7 0 0 0 0 0

16 14 0 0 0 0 0 2 6 0 0 0 1 0 6 0 0 3 0 1 3 5 1 8 0 0 0 0 0

15 15 0 0 0 1 0 0 5 3 0 0 0 0 8 1 0 0 0 0 1 8 4 0 1 0 2 2 0

14 16 1 0 0 4 0 1 0 1 2 0 1 0 8 1 0 0 0 1 5 3 0 0 0 0 5 3 1

13 17 1 0 0 0 3 0 0 3 0 3 0 0 6 3 0 0 0 0 6 3 3 0 5 0 0 1 0

12 18 0 0 0 1 1 1 3 2 0 0 1 0 6 1 0 2 0 1 1 7 6 3 0 0 0 0 0

20 11 0 0 0 0 1 0 0 6 0 0 2 0 7 0 0 0 2 2 7 0 0 9 0 0 0 0 0

17 14 0 0 0 1 0 0 4 2 0 0 2 0 7 0 0 2 0 2 2 5 4 5 0 0 0 0 0

16 15 0 0 0 0 0 0 5 4 0 0 0 0 4 3 0 2 0 4 5 0 5 0 3 0 1 0 0

15 16 1 0 2 6 1 0 0 0 0 0 0 0 6 0 0 3 0 3 5 1 0 0 0 0 7 2 0

14 17 1 0 3 3 3 0 0 0 0 0 0 0 5 0 0 4 0 2 4 3 0 0 0 1 3 5 0

13 18 0 1 0 2 4 0 0 0 0 2 1 0 8 0 0 1 0 1 4 4 4 0 4 1 0 0 0

12 19 1 1 0 0 2 0 1 5 0 1 0 0 7 2 0 0 0 2 3 4 6 0 1 2 0 0 0

18 14 0 0 0 0 2 0 1 0 0 0 6 0 3 0 0 4 2 6 2 1 9 0 0 0 0 0 0

17 15 0 0 0 0 0 0 0 9 0 0 0 0 7 0 0 2 0 0 0 9 6 0 0 0 3 0 0

16 16 1 0 0 0 3 0 2 4 0 0 0 0 6 3 0 0 0 0 3 6 0 0 0 0 1 3 5

15 17 1 0 0 2 3 0 0 1 0 2 1 0 4 3 0 2 0 1 4 4 0 0 0 0 0 7 2

14 18 0 0 2 2 2 0 0 0 0 2 0 1 4 1 0 4 0 1 0 8 6 1 2 0 0 0 0

13 19 0 1 3 5 0 0 0 0 0 0 1 0 4 2 0 3 0 4 4 1 0 9 0 0 0 0 0

12 20 1 1 0 0 3 1 2 2 0 0 1 0 5 3 0 1 0 1 7 1 1 2 5 1 0 0 0

18 15 0 0 0 1 0 0 2 3 0 1 1 1 5 3 0 1 0 2 3 4 4 2 3 0 0 0 0

17 16 1 0 0 0 1 1 5 2 0 0 0 0 8 0 0 1 0 0 1 8 7 0 2 0 0 0 0

16 17 1 0 1 0 1 1 2 3 0 0 1 0 6 1 0 2 0 1 1 7 0 0 0 0 1 8 0

15 18 1 0 1 4 2 0 0 1 0 1 0 0 5 3 0 1 0 1 2 6 2 0 2 0 0 5 0

14 19 0 0 5 2 0 0 0 1 0 1 0 0 9 0 0 0 0 0 3 6 3 3 3 0 0 0 0

13 20 0 0 0 0 0 5 2 2 0 0 0 0 7 1 0 1 0 0 7 2 3 5 1 0 0 0 0

19 15 0 0 0 1 1 0 5 1 0 0 1 0 7 2 0 0 0 1 1 7 7 2 0 0 0 0 0

18 16 0 0 0 0 2 0 1 5 0 0 1 0 8 0 0 1 0 1 0 8 6 1 2 0 0 0 0

17 17 0 0 0 0 0 3 2 3 0 0 1 0 7 0 0 2 0 1 3 5 7 0 0 0 2 0 0

16 18 0 0 4 3 2 0 0 0 0 0 0 0 7 0 0 2 0 0 2 7 5 0 4 0 0 0 0

15 19 1 1 2 3 3 0 0 1 0 0 0 0 4 3 0 2 0 2 7 0 3 0 6 0 0 0 0

14 20 0 0 1 4 0 0 2 0 0 0 2 0 6 1 0 1 1 2 2 5 0 9 0 0 0 0 0

19 16 0 0 0 0 0 3 2 3 0 0 1 0 7 2 0 0 0 1 6 2 0 9 0 0 0 0 0

18 17 0 1 0 0 1 0 3 3 0 0 2 0 4 0 0 5 0 4 1 4 7 1 1 0 0 0 0

17 18 1 0 0 0 6 0 1 0 0 0 2 0 7 2 0 0 0 2 4 3 3 1 3 1 1 0 0

16 19 0 0 0 0 2 1 0 5 0 0 1 0 7 0 0 2 0 1 6 2 4 0 5 0 0 0 0

20 16 0 0 0 1 0 0 5 2 1 0 0 0 8 0 0 1 0 1 6 2 2 7 0 0 0 0 0

19 17 0 0 0 1 5 0 0 2 0 0 1 0 7 0 0 1 1 2 4 3 4 5 0 0 0 0 0

18 18 1 1 0 0 0 0 2 3 0 4 0 0 7 1 0 1 0 0 3 6 7 0 2 0 0 0 0

17 19 0 0 5 0 0 0 0 2 0 2 0 0 8 0 0 1 0 3 6 0 5 4 0 0 0 0 0

16 20 0 1 1 0 2 0 0 2 0 3 1 0 0 3 0 1 0 3 6 0 4 5 0 0 0 0 0

19 18 0 0 0 0 4 0 5 0 0 0 0 0 7 0 0 2 0 0 9 0 4 5 0 0 0 0 0

18 19 1 0 0 0 0 0 0 5 0 4 0 0 4 3 0 2 0 0 3 6 4 1 4 0 0 0 0

17 20 0 0 0 0 0 4 0 0 0 4 1 0 6 0 0 3 0 1 7 1 4 5 0 0 0 0 0

NA NA 1 1 1 3 1 0 0 1 0 2 1 0 5 2 0 1 1 1 3 5 0 2 5 2 0 0 0

NA NA 0 1 0 0 2 0 0 0 0 7 0 0 2 0 0 4 3 7 2 0 0 9 0 0 0 0 0

NA NA 1 1 0 0 4 0 0 0 0 0 5 0 2 0 1 2 4 5 4 0 0 1 6 2 0 0 0

NA NA 0 1 0 5 0 0 2 0 0 0 2 0 8 0 0 1 0 2 7 0 2 0 3 1 3 0 0

NA NA 0 1 0 0 0 0 0 0 0 0 9 0 0 0 0 4 5 9 0 0 0 8 1 0 0 0 0

NA NA 0 1 0 0 0 0 3 3 0 0 3 0 4 4 0 1 0 3 6 0 3 0 2 1 3 0 0

NA NA 1 1 0 5 1 0 0 0 0 0 3 0 4 0 0 5 0 3 4 2 0 2 2 0 5 0 0

NA NA 1 1 0 0 0 0 3 2 2 0 2 0 3 0 0 6 0 2 7 0 2 0 5 0 2 0 0

NA NA 1 1 0 5 4 0 0 0 0 0 0 0 8 0 0 1 0 0 7 2 0 0 4 1 3 1 0

NA NA 1 1 0 5 3 0 0 0 0 1 0 0 5 3 0 1 0 3 4 2 0 1 5 0 3 0 0

NA NA 1 1 0 0 1 0 0 2 0 6 0 0 4 0 0 5 0 0 2 7 0 2 6 1 0 0 0

NA NA 0 1 0 0 1 0 0 8 0 0 0 0 8 1 0 0 0 0 9 0 5 2 1 1 0 0 0

NA NA 0 1 0 0 1 0 0 4 4 0 0 0 8 0 0 1 0 0 0 9 5 0 4 0 0 0 0

NA NA 1 1 0 1 5 0 0 0 0 0 0 0 9 0 0 0 0 0 6 3 2 1 5 1 0 0 0

NA NA 1 1 0 2 1 0 0 5 0 0 1 0 5 1 0 3 0 1 5 3 1 0 6 0 1 1 0

NA NA 0 1 0 3 6 0 0 0 0 0 0 0 8 1 0 0 0 0 5 4 4 5 0 0 0 0 0

NA NA 0 1 0 5 0 0 2 1 0 0 1 0 5 1 0 3 0 1 8 0 2 7 0 0 0 0 0

NA NA 1 1 2 0 5 1 0 1 0 0 0 0 5 1 0 3 0 0 5 4 1 4 3 1 0 0 0

NA NA 0 1 0 3 3 0 0 0 0 0 3 0 5 0 0 1 3 3 4 2 1 2 5 1 0 0 0

NA NA 0 1 0 4 4 0 0 0 0 0 1 0 6 2 0 0 1 2 5 2 2 7 0 0 0 0 0

NA NA 1 1 0 1 0 0 0 5 0 2 1 0 6 2 0 0 1 3 6 0 1 0 8 0 0 0 0

NA NA 0 1 0 1 0 0 6 0 0 0 2 0 6 0 0 3 0 2 0 7 5 3 1 0 0 0 0

NA NA 0 1 0 0 6 0 1 0 0 0 2 0 4 1 0 4 0 2 5 2 2 7 0 0 0 0 0

NA NA 0 1 0 5 1 0 0 1 0 0 2 0 6 1 0 2 0 2 6 1 4 5 0 0 0 0 0

NA NA 0 1 0 4 1 0 0 0 0 0 4 0 5 0 0 0 4 4 5 0 0 9 0 0 0 0 0

NA NA 0 1 0 0 0 1 3 2 1 1 1 0 7 2 0 0 0 1 2 6 1 7 1 0 0 0 0

NA NA 0 1 0 4 5 0 0 0 0 0 0 0 8 0 0 1 0 9 0 0 1 8 0 0 0 0 0

NA NA 1 1 0 2 4 2 0 0 0 0 1 0 8 0 0 1 0 1 8 0 6 3 0 0 0 0 0

NA NA 0 1 0 0 3 1 2 3 0 0 0 0 7 2 0 0 0 2 5 2 4 0 4 1 0 0 0

NA NA 0 1 0 0 3 1 2 1 0 0 2 0 6 3 0 0 0 0 3 6 3 0 6 0 0 0 0

NA NA 0 1 0 0 1 0 0 5 0 3 0 0 3 3 0 3 0 1 6 2 4 0 0 4 1 0 0

NA NA 0 1 2 0 0 0 0 5 0 0 2 0 7 0 0 2 0 2 3 4 4 5 0 0 0 0 0

NA NA 0 1 3 0 3 0 0 3 0 0 0 0 0 1 0 2 0 1 2 6 6 3 0 0 0 0 0

NA NA 0 1 4 3 2 0 0 0 0 0 0 0 8 0 0 1 0 2 3 4 0 1 8 0 0 0 0

NA NA 0 1 0 1 2 0 0 5 0 0 1 0 8 1 0 0 0 1 8 0 3 6 0 0 0 0 0

NA NA 0 1 0 3 5 0 0 1 0 0 0 0 9 0 0 0 0 0 8 1 6 1 2 0 0 0 0
