# Supplementary material for: Combining Niche and Dispersal in a Simple Model (NDM) of Species Distribution
Source: PLoS One. 2013 Nov 12;8(11):e79948. doi: 10.1371/journal.pone.0079948 (PMC3827172; doi:10.1371/journal.pone.0079948)
Supplement: Dataset S2 — Presence-absence data of breeding bird communities and corresponding habitat variables in the Vanera valley (French Eastern Pyrenees, 42° 23′ N, 2° E) in 1982. (DOC) [file pone.0079948.s004.doc]

**Dataset S2**. Presence-absence data of breeding bird communities and corresponding habitat variables in the Vanera valley (French Eastern Pyrenees, 42° 23’ N, 2° E) in 1982.

***Description of columns:***

*Code is to do the correspondence with dataset S3: code of 214 cells out of 302, to map the corresponding data on a grid (19 lines and 24 columns with line and column numbers indicated in dataset S3). For the other cells, code is unavailable (NA).*

*Next 45 columns are abbreviations of bird species (absence=0, presence=1):*

PMA Parus major

CCE Cyanistes caeruleus

PAT Periparus ater PAT

LCR Lophophanes cristatus LCR

CBR Certhia brachydactyla

TTR Troglodytes troglodytes

TVI Turdus viscivorus

TPH Turdus philomelos

TME Turdus merula

SRB Saxicola rubicola SRB

LME Luscinia megarhynchos

ERU Erithacus rubecula

SAT Sylvia atricapilla

SBO Sylvia borin

SCO Sylvia communis

PCO Phylloscopus collybita

PBO Phylloscopus bonelli

RRE Regulus regulus

PMO Prunella modularis

ATR Anthus trivialis

CCA Carduelis carduelis

CCN Carduelis cannabina

SSE Serinus serinus

PPY Pyrrhula pyrrhula

FCO Fringilla coelebs

ECI Emberiza citrinella

AAR Alauda arvensis

TTO Turdus torquatus

SRU Saxicola rubetra

SCI Serinus citrinella

LCU Loxia curvirostra

ECI Emberiza cia

POC Phoenicurus ochruros

LAR Lullula arborea

ASP Anthus spinoletta

ECA Emberiza calandra

OOE Oenanthe oenanthe

PCL Prunella collaris

DMA Dendrocopos major

JTO Jynx torquilla

PVI Picus viridis

UEP Upupa epops

CCO Coturnix coturnix

CPA Columba palumbus

CCR Cuculus canorus

*Remaining columns : Altitude (m), Pine forest (boolean : yes=1, no=0), Herbac=Herbaceous plant cover (%), Lig01=Ligneous plant cover 0-1m (%), Lig14=Ligneous plant cover 1-4m (%),Lig8=Ligneous plant cover >8m (%).*

Code PMA CEE PAT LCR CBR TTR TVI TPH TME SRB LME ERU SAT SBO SCO PCO PBO RRE PMO ATR CCA CCN SSE PPY FCO ECI AAR TTO SRU SCI LCU ECI POC LAR ASP ECA OOE PCL DMA JTO PVI UEP CCO CPA CCR Altitude Pine Herbac Lig01 Lig14 Lig8

1EA 0 1 0 0 0 0 0 0 1 0 1 0 0 0 1 0 0 0 0 0 0 0 0 0 0 0 1 0 0 0 0 0 0 0 0 1 0 0 0 0 0 0 0 1 0 1170 1 87.5 12.5 12.5 12.5

1EB 0 0 0 0 0 0 0 0 0 0 0 0 0 0 0 0 0 0 0 0 0 0 0 0 0 0 1 0 0 0 0 0 0 0 0 1 0 0 0 0 0 0 1 0 0 1185 1 87.5 0 0 12.5

1EC 0 0 0 0 0 0 0 0 0 0 0 0 0 0 0 0 0 0 0 0 0 0 0 0 0 0 1 0 1 0 0 0 0 0 0 1 0 0 0 0 0 0 1 0 1 1190 1 87.5 0 0 0

1ED 0 0 0 0 0 0 0 0 1 0 1 0 0 0 0 0 0 0 0 0 0 0 0 0 1 1 1 0 0 0 0 0 0 0 0 1 0 0 0 1 1 1 1 0 0 1170 1 87.5 37.5 37.5 37.5

1EE 1 1 0 0 1 1 0 0 0 0 1 0 1 0 0 0 0 0 0 0 0 0 1 0 1 1 1 0 0 0 0 0 0 0 0 0 0 0 0 0 1 0 0 0 1 1160 1 62.5 12.5 37.5 37.5

1EF 1 1 0 0 0 0 0 0 1 0 1 0 1 0 1 0 0 0 0 0 0 0 1 0 0 0 1 0 0 0 0 0 0 0 0 1 0 0 0 0 1 0 0 0 1 1200 1 62.5 37.5 37.5 0

NA 0 0 1 0 0 0 0 0 1 0 0 1 0 0 0 0 0 1 1 0 0 0 0 0 1 0 0 0 0 1 1 0 0 0 0 0 0 0 0 0 0 0 0 0 0 1450 2 62.5 37.5 0 87.5

NA 0 0 1 1 0 0 1 0 0 0 0 0 0 1 1 0 0 1 1 0 0 0 0 0 1 0 0 0 0 0 0 0 0 0 0 0 0 0 0 0 0 0 0 1 0 1500 2 37.5 37.5 0 87.5

1FA 1 0 0 0 0 0 0 0 0 0 1 0 1 1 1 0 0 0 0 0 0 0 0 0 0 0 1 0 0 0 0 0 0 0 0 0 0 0 0 0 0 1 1 0 0 1180 1 62.5 37.5 37.5 37.5

1FB 0 0 0 0 0 0 0 0 0 0 1 0 0 0 0 0 0 0 0 0 0 0 0 0 0 0 1 0 0 0 0 0 0 0 0 1 0 0 0 0 0 0 1 0 1 1200 1 87.5 12.5 12.5 0

1FC 0 0 0 0 0 0 0 0 0 0 0 0 0 0 0 0 0 0 0 0 0 0 0 0 0 0 1 0 0 0 0 0 0 0 0 1 1 0 0 0 0 1 1 0 1 1200 1 87.5 0 0 12.5

1FE 1 1 0 0 1 1 0 0 0 0 1 0 1 0 1 0 0 0 0 0 0 0 1 0 1 1 1 0 0 0 0 0 0 0 0 0 0 0 0 0 1 0 0 0 1 1180 1 87.5 12.5 12.5 37.5

1FF 1 0 0 0 0 0 0 0 1 1 0 0 1 0 0 0 1 0 0 0 0 1 1 0 1 0 1 0 0 0 0 0 0 0 0 0 0 0 0 1 0 0 0 0 1 1270 1 87.5 12.5 12.5 12.5

1FG 1 0 1 1 1 0 0 0 1 0 0 0 1 1 1 1 1 0 1 1 0 1 1 0 1 0 1 0 0 0 0 0 0 0 0 0 0 0 0 0 0 0 0 0 1 1320 1 37.5 37.5 37.5 37.5

1FH 1 0 1 1 0 0 0 0 1 0 0 0 1 0 0 0 0 1 1 0 0 0 0 0 1 0 0 0 0 0 0 0 0 0 0 0 0 0 0 0 1 0 0 0 0 1440 2 37.5 12.5 37.5 87.5

1FI 0 0 1 1 1 0 0 1 1 0 0 1 0 0 0 0 0 1 0 0 0 0 0 0 1 0 0 0 0 0 0 0 0 0 0 0 0 0 0 0 0 0 0 0 1 1530 2 50 0 0 87.5

1FJ 0 0 1 0 0 0 0 1 1 0 0 1 0 0 0 0 0 0 0 0 0 0 0 0 1 0 0 0 0 0 0 0 0 0 0 0 0 0 0 0 0 0 0 1 1 1600 2 62.5 12.5 0 87.5

1FK 0 0 1 0 1 0 0 0 0 0 0 1 0 0 0 0 0 1 0 0 0 0 0 0 1 0 0 0 0 0 0 0 0 0 0 0 0 0 0 0 0 0 0 0 0 1640 2 62.5 0 0 87.5

NA 0 0 1 1 0 0 0 0 0 0 0 1 0 0 0 0 0 1 0 0 0 0 0 1 1 0 0 0 0 0 1 0 0 0 0 0 0 0 1 0 0 0 0 0 0 1720 2 87.5 12.5 0 37.5

NA 0 0 1 1 1 0 1 1 0 0 0 1 0 0 0 0 0 1 1 1 0 0 0 1 1 0 0 1 0 1 0 0 0 0 0 0 0 0 1 0 0 0 0 0 0 1740 2 87.5 87.5 87.5 12.5

1GA 1 0 0 0 1 0 0 0 0 0 1 1 1 1 0 0 0 0 0 1 0 0 1 0 1 1 0 0 0 0 0 0 0 0 0 0 0 0 0 0 0 0 0 0 0 1180 1 87.5 37.5 37.5 37.5

1GB 0 0 0 0 0 0 0 0 0 0 0 0 0 0 1 0 0 0 0 1 1 0 0 0 1 0 1 0 0 0 0 0 0 0 0 0 0 0 0 0 1 0 0 0 1 1210 1 87.5 37.5 37.5 12.5

1GE 1 0 0 0 1 0 0 0 0 0 1 0 1 0 0 0 0 0 0 0 0 0 1 0 1 0 1 0 0 0 0 0 0 0 0 0 0 0 0 0 0 0 0 0 0 1190 1 87.5 12.5 12.5 12.5

1GF 1 1 0 0 1 0 0 0 1 0 1 0 1 0 1 0 0 0 0 0 0 0 0 0 1 0 0 0 0 0 0 0 0 0 0 0 0 0 0 0 0 0 0 0 1 1220 1 75 37.5 37.5 12.5

1GG 1 0 1 0 0 1 0 0 1 0 1 0 1 0 0 0 1 0 1 0 0 0 0 1 1 0 1 0 0 0 0 1 0 0 0 0 0 0 0 0 0 0 0 0 1 1280 1 37.5 12.5 37.5 12.5

1GH 1 0 1 1 0 0 0 0 1 0 0 1 1 0 0 0 0 0 1 0 0 0 0 0 1 0 1 0 0 0 0 0 0 0 0 0 0 0 0 0 0 0 0 0 1 1360 1 62.5 12.5 37.5 0

1GI 0 0 1 1 0 0 0 0 1 0 0 0 0 0 1 1 1 0 1 0 0 0 0 0 1 0 1 0 0 0 0 1 0 0 0 0 0 0 0 0 0 0 0 0 1 1400 1 12.5 62.5 37.5 37.5

1GJ 1 0 1 1 0 0 0 0 1 0 0 1 1 0 1 0 0 0 1 0 0 0 0 1 1 0 0 0 0 0 0 0 0 0 0 0 0 0 0 0 0 0 0 0 1 1570 1 37.5 87.5 37.5 37.5

1GK 0 0 1 1 1 0 0 0 1 0 0 1 0 0 0 0 0 0 0 0 0 0 0 1 1 0 0 0 0 0 0 0 0 0 0 0 0 0 0 0 0 0 0 0 1 1630 2 87.5 0 0 37.5

1GL 0 0 1 1 1 0 0 0 0 0 0 0 0 0 0 0 0 0 1 0 0 0 0 0 1 0 0 0 0 1 0 0 0 0 0 0 0 0 1 0 0 0 0 0 1 1720 2 87.5 37.5 62.5 50

1GM 0 0 1 1 0 0 0 1 1 0 0 1 0 0 0 0 0 1 0 0 0 0 0 1 1 0 0 0 0 1 0 0 0 0 0 0 0 0 0 0 0 0 0 0 1 1780 2 87.5 12.5 37.5 12.5

NA 0 0 1 1 0 0 0 0 0 0 0 1 0 0 1 0 0 1 0 0 0 0 0 0 1 0 0 0 0 1 0 0 0 0 0 0 0 0 0 0 0 0 0 0 0 1600 2 62.5 43.75 0 43.75

NA 0 0 1 0 0 1 1 0 0 0 0 1 0 0 0 0 0 0 0 0 0 0 0 1 1 0 0 0 0 1 0 0 0 0 0 0 0 0 0 0 0 0 0 0 0 1630 2 62.5 12.5 18.75 37.5

NA 0 0 1 0 0 0 1 1 0 0 0 1 0 1 0 0 0 1 1 0 0 0 0 0 1 0 0 0 0 0 1 0 0 0 0 0 0 0 0 0 0 0 0 0 0 1740 2 75 37.5 37.5 12.5

NA 0 0 1 1 0 0 0 1 0 0 0 1 0 0 0 0 0 0 1 0 0 0 0 0 1 0 0 1 0 1 1 0 0 1 0 0 0 0 0 0 0 0 0 0 0 1820 2 87.5 18.75 18.75 50

1HA 0 0 0 0 0 0 0 0 1 0 1 0 0 0 1 0 0 0 0 0 0 0 0 0 0 1 1 0 0 0 0 0 0 0 0 0 0 0 0 0 1 0 0 0 0 1200 1 87.5 37.5 37.5 0

1HB 0 0 0 0 0 0 0 0 1 0 1 0 1 0 0 0 0 0 0 1 0 0 1 0 0 1 1 0 1 0 0 0 0 0 0 0 0 0 0 1 0 0 0 0 1 1215 1 87.5 12.5 37.5 37.5

1HE 1 0 0 0 1 1 0 0 0 0 1 0 1 0 0 0 0 0 0 0 0 0 1 0 1 0 0 0 0 0 0 0 0 0 0 0 0 0 0 0 0 1 0 0 0 1200 1 62.5 12.5 12.5 37.5

1HF 1 0 0 0 0 0 0 0 1 1 0 0 1 1 0 0 0 0 1 0 0 0 1 0 1 1 0 0 0 0 0 1 0 0 0 0 0 0 0 1 1 0 0 0 1 1260 1 50 12.5 12.5 12.5

1HG 0 0 0 0 0 0 0 0 1 0 0 0 1 0 1 0 0 0 0 0 0 1 0 0 1 0 1 0 0 0 0 0 0 0 0 0 0 0 0 0 1 0 0 0 1 1400 1 87.5 31.25 0 6.25

1HH 1 0 1 0 0 0 0 0 1 0 0 0 1 0 0 0 1 0 0 0 0 0 0 1 1 0 1 0 0 0 0 0 0 0 0 0 0 0 0 0 0 0 0 0 1 1380 1 87.5 12.5 12.5 12.5

1HI 1 0 1 0 0 1 0 0 0 0 0 1 0 1 0 1 1 0 1 0 0 0 0 0 1 0 1 0 0 0 0 0 0 0 0 0 0 0 0 0 0 0 0 0 1 1400 1 43.75 12.5 37.5 12.5

1HJ 0 0 1 0 0 1 0 0 1 0 0 0 0 0 0 0 0 1 0 0 0 0 0 0 1 0 0 0 0 0 0 0 0 0 0 0 0 0 0 0 1 0 0 0 0 1440 1 43.75 0 12.5 37.5

1HK 0 0 1 1 1 1 0 1 0 0 0 1 1 1 0 0 0 1 1 0 0 0 0 1 0 0 0 0 0 0 0 0 0 0 0 0 0 0 1 0 0 0 0 0 0 1560 2 87.5 12.5 12.5 62.5

NA 0 0 1 0 0 1 0 0 0 0 0 1 0 0 0 0 0 0 0 0 0 0 0 1 1 0 0 0 0 0 0 0 0 0 0 0 0 0 0 0 0 0 0 0 0 1560 2 87.5 37.5 37.5 12.5

1HL 0 0 1 1 0 1 0 1 1 0 0 1 0 0 0 0 0 1 1 0 0 0 0 0 1 0 0 0 0 0 0 0 0 0 0 0 0 0 1 0 0 0 0 0 1 1650 2 87.5 62.5 87.5 0

1HM 0 0 1 1 0 1 0 1 1 0 0 1 0 0 0 0 0 1 0 0 0 0 0 0 1 0 0 0 0 1 1 0 0 0 0 0 0 0 1 0 0 0 0 0 1 1770 2 87.5 12.5 12.5 12.5

NA 0 0 1 0 0 0 1 0 0 0 0 1 0 0 0 0 0 1 0 0 0 0 0 0 1 0 0 0 0 0 0 0 0 0 0 0 0 0 1 0 0 0 0 0 0 1500 2 87.5 62.5 0 87.5

NA 0 0 1 0 0 1 0 1 0 0 0 1 0 0 0 0 0 1 0 0 0 0 0 1 1 0 0 0 0 0 0 0 0 0 0 0 0 0 1 0 0 0 0 0 0 1600 2 87.5 0 6.25 62.5

NA 0 0 1 0 0 0 1 0 0 0 0 0 0 1 0 0 0 1 1 1 0 0 0 1 1 0 0 0 0 0 1 0 0 0 0 0 0 0 0 0 0 0 0 0 0 1710 2 87.5 37.5 18.75 31.25

NA 0 0 1 1 1 1 0 1 0 0 0 1 0 0 0 0 0 1 0 0 0 0 0 0 1 0 0 0 0 1 0 0 0 0 0 0 0 0 0 0 0 0 0 1 0 1830 2 87.5 12.5 12.5 37.5

1IA 0 0 0 0 0 0 0 0 1 0 1 0 0 0 1 0 0 0 1 0 0 1 0 0 0 0 1 0 0 0 0 0 0 0 0 0 0 0 0 0 1 0 0 0 1 1200 1 87.5 12.5 0 12.5

1IB 0 0 0 0 0 0 0 0 1 0 1 0 0 0 0 0 0 0 0 0 0 0 0 0 0 1 1 0 0 0 0 0 0 0 0 0 1 0 0 0 0 0 0 0 1 1240 1 87.5 0 0 6.25

1IC 1 1 0 0 0 1 0 0 1 1 1 0 1 1 1 0 1 0 1 1 0 1 1 0 1 0 1 0 0 0 0 0 0 0 0 0 0 0 0 0 0 0 0 0 0 1260 1 37.5 12.5 12.5 12.5

1IE 1 0 1 0 1 0 0 0 0 0 1 0 1 0 0 0 0 0 0 0 1 0 0 0 1 0 0 0 0 1 0 0 1 0 0 0 0 0 0 0 0 1 0 0 1 1220 1 37.5 12.5 37.5 37.5

1IF 1 1 0 0 0 0 0 0 1 0 0 0 1 1 0 0 1 0 0 1 1 0 1 1 1 0 0 0 0 0 0 1 0 0 0 0 0 0 0 0 0 0 0 0 1 1280 1 62.5 62.5 37.5 18.75

1IG 1 0 0 0 0 0 0 0 1 0 0 1 1 0 1 1 0 0 1 0 0 0 0 0 1 0 1 0 0 0 0 0 0 0 0 0 0 0 0 0 0 0 0 0 1 1420 1 87.5 87.5 50 0

1IH 0 0 1 1 0 0 0 0 1 0 0 0 0 0 0 0 0 0 1 0 0 0 0 0 0 0 1 0 0 1 0 0 0 0 0 0 0 0 0 0 1 0 0 0 0 1530 1 62.5 37.5 12.5 0

1II 1 0 1 1 0 1 1 0 1 0 0 1 1 0 0 1 1 1 1 0 0 0 0 0 0 0 0 0 0 1 0 0 0 0 0 0 0 0 0 0 0 0 0 0 1 1540 2 62.5 62.5 12.5 0

1IJ 0 0 1 1 0 0 0 0 1 0 0 1 0 0 0 0 1 1 0 0 0 0 0 0 0 0 1 0 0 1 0 0 0 0 0 0 0 0 0 0 0 0 0 0 1 1600 2 50 0 12.5 87.5

1IK 0 0 1 1 1 0 0 0 0 0 0 1 0 0 0 0 0 0 1 0 0 0 0 1 1 0 0 0 0 0 1 0 0 0 0 0 0 0 1 0 0 0 0 0 0 1640 2 62.5 0 0 87.5

1IL 0 0 1 1 0 1 0 1 0 0 0 1 0 0 0 0 0 1 1 0 0 0 0 1 1 0 0 0 0 0 1 0 0 0 0 0 0 0 0 0 0 0 0 0 0 1700 2 62.5 18.75 18.75 12.5

1IM 0 0 1 1 0 1 0 0 0 0 0 0 0 0 0 0 0 0 0 0 0 0 0 0 1 0 0 1 0 1 1 0 0 0 0 0 0 0 0 0 0 0 0 0 0 1770 2 62.5 12.5 0 12.5

1IN 0 0 1 1 0 0 0 1 0 0 0 1 0 0 0 0 0 1 0 0 0 0 0 0 1 0 0 0 0 1 1 0 0 0 0 0 0 0 0 0 0 0 0 0 0 1880 2 87.5 12.5 0 12.5

1IO 0 0 1 0 0 0 0 0 1 0 0 1 0 0 0 0 0 0 1 0 0 0 0 0 1 0 1 0 0 1 1 0 0 0 0 0 0 0 0 0 0 0 0 0 0 1930 2 87.5 50 43.75 43.75

NA 0 0 1 0 0 0 0 0 0 0 0 1 0 0 0 1 0 0 0 0 0 0 0 0 1 0 0 0 0 0 0 0 0 0 0 0 0 0 0 0 0 0 0 0 0 1640 2 12.5 87.5 87.5 0

NA 0 0 1 1 1 0 1 0 0 0 0 1 0 0 0 0 0 1 0 0 0 0 0 1 1 0 0 0 0 0 1 0 0 0 0 0 0 0 1 0 0 0 0 0 0 1700 2 87.5 18.75 31.25 87.5

NA 0 0 1 1 1 1 1 0 0 0 0 1 0 0 0 0 0 0 0 1 0 0 0 1 1 0 0 0 0 1 1 0 0 0 0 0 0 0 1 0 0 0 0 0 0 1750 2 87.5 37.5 37.5 87.5

NA 0 0 1 0 1 1 0 1 0 0 0 1 0 0 0 0 0 1 1 0 0 0 0 1 1 0 0 0 0 0 1 0 0 0 0 0 0 0 0 0 0 0 0 0 0 1770 2 87.5 37.5 12.5 12.5

NA 0 0 0 0 1 1 0 0 0 0 0 1 0 0 0 0 0 1 0 0 0 0 0 0 1 0 0 0 0 1 1 0 0 0 0 0 0 0 0 0 0 0 0 0 0 1800 2 87.5 43.75 6.25 37.5

NA 0 0 1 1 0 0 0 0 1 0 0 1 0 0 0 0 0 0 1 0 0 0 0 0 1 0 1 0 0 1 1 0 0 0 0 0 0 0 0 0 0 0 0 0 0 1900 2 87.5 0 18.75 6.25

1JA 0 0 0 0 0 0 0 0 1 0 1 0 0 1 0 0 0 0 0 0 0 1 0 0 0 0 1 0 0 0 0 0 0 0 0 0 0 0 0 0 1 0 0 0 0 1240 1 87.5 37.5 37.5 12.5

1JB 1 0 0 0 0 0 0 0 1 0 1 0 1 0 0 0 0 0 0 0 1 0 0 0 0 0 1 0 0 0 0 0 0 0 0 0 0 0 0 0 0 0 0 1 0 1260 1 87.5 37.5 37.5 37.5

1JC 0 0 0 0 0 0 0 0 1 0 1 0 1 1 1 0 1 0 0 1 0 0 1 0 0 0 1 0 0 0 0 0 0 0 0 0 0 0 0 0 1 0 0 0 1 1300 1 87.5 12.5 12.5 37.5

1JD 1 1 0 0 0 0 0 0 1 0 1 0 1 0 0 0 1 0 0 0 0 0 0 0 0 0 1 0 0 0 0 0 0 0 0 0 0 0 0 0 1 1 0 0 1 1300 1 87.5 12.5 37.5 37.5

1JE 1 0 0 0 0 0 0 0 1 0 1 0 1 0 0 0 0 0 0 0 1 0 1 0 1 0 0 0 0 0 0 0 1 0 0 0 0 0 0 1 0 0 0 0 1 1260 1 87.5 12.5 37.5 37.5

1JF 1 0 0 0 0 0 0 0 1 0 1 0 0 1 0 0 0 0 1 0 1 0 0 0 0 0 0 0 0 1 0 0 0 0 0 0 0 0 0 1 0 0 0 0 1 1300 1 62.5 62.5 62.5 0

1JG 1 0 1 0 0 0 0 0 1 1 0 0 1 1 1 0 1 0 1 0 0 0 0 0 1 0 0 0 0 1 0 0 0 0 0 0 0 0 0 0 0 0 0 0 0 1360 1 62.5 87.5 12.5 0

1JH 1 0 1 1 1 0 0 0 1 0 0 1 0 0 0 0 0 0 1 1 0 0 0 0 1 0 0 0 0 1 0 0 0 1 0 0 0 0 0 0 0 0 0 0 1 1500 2 87.5 62.5 37.5 37.5

1JI 0 0 1 1 0 1 0 0 1 0 0 0 0 0 1 0 0 1 1 0 0 0 0 0 1 0 1 0 0 0 0 0 0 0 0 0 0 0 0 0 0 0 0 0 1 1610 2 43.75 12.5 0 62.5

1JJ 0 0 1 0 0 0 0 0 0 0 0 1 0 0 0 0 0 1 0 0 0 0 0 0 1 0 0 0 0 1 0 0 0 0 0 0 0 0 0 0 0 0 0 0 0 1680 2 87.5 0 0 87.5

1JK 0 0 1 0 1 0 0 0 1 0 0 0 0 0 0 0 0 0 1 0 0 0 0 0 1 0 0 0 0 0 1 0 0 0 0 0 0 0 0 0 0 0 0 0 0 1780 2 87.5 37.5 37.5 12.5

1JL 0 0 1 0 1 0 0 0 1 0 0 0 0 0 0 0 0 1 1 0 0 0 0 0 1 0 0 0 0 1 1 0 0 0 0 0 0 0 1 0 0 0 0 0 0 1800 2 87.5 12.5 12.5 37.5

1JM 0 0 1 1 1 1 0 0 0 0 0 1 0 0 0 0 0 1 0 0 0 0 0 1 1 0 0 0 0 0 0 0 0 0 0 0 0 0 0 0 0 0 0 0 0 1820 2 62.5 37.5 37.5 12.5

1JN 0 0 1 1 1 1 0 1 0 0 0 1 0 0 0 0 0 1 0 0 0 0 0 0 1 0 0 0 0 1 0 0 0 0 0 0 0 0 0 0 0 0 0 0 0 1900 2 87.5 12.5 0 37.5

1JO 0 0 1 0 0 0 0 1 0 0 0 0 0 0 0 0 0 0 0 0 0 0 0 0 1 0 1 0 0 1 0 0 0 0 0 0 0 0 0 0 0 0 0 0 0 1990 1 87.5 0 0 0

1JP 0 0 0 0 0 0 0 0 0 0 0 0 0 0 0 0 0 0 0 0 0 0 0 0 0 0 1 0 0 0 0 0 0 1 0 0 0 0 0 0 0 0 0 0 0 2000 1 87.5 0 0 0

NA 0 0 1 1 0 1 1 0 0 0 0 1 0 0 0 0 0 1 0 0 0 0 0 0 1 1 0 0 0 0 0 0 0 0 0 0 0 0 1 0 0 0 0 1 0 1600 2 87.5 62.5 12.5 87.5

NA 0 0 1 1 0 0 1 0 0 0 0 1 0 0 0 0 0 0 1 1 0 0 0 0 1 0 0 0 0 1 1 0 0 1 0 0 0 0 0 0 0 0 0 0 0 1740 2 87.5 87.5 62.5 12.5

NA 0 0 1 1 0 0 0 0 0 0 0 1 0 0 0 0 0 0 1 1 0 0 0 0 1 0 0 0 0 1 0 0 0 0 0 0 0 0 0 0 0 0 0 0 0 1840 2 87.5 62.5 62.5 31.25

NA 0 0 1 0 0 1 0 0 0 0 0 1 0 0 0 0 0 0 1 0 0 0 0 1 0 0 0 0 0 0 0 0 0 0 0 0 0 0 1 0 0 0 0 0 0 1820 2 87.5 62.5 37.5 12.5

NA 0 0 0 0 0 0 0 1 0 0 0 1 0 0 0 0 0 1 1 0 0 0 0 0 1 0 1 1 0 1 0 0 0 0 0 0 0 0 0 0 0 0 0 0 0 1940 2 87.5 12.5 37.5 37.5

1KA 0 0 0 0 0 0 0 0 1 0 1 0 0 0 1 0 0 0 1 0 0 0 1 0 0 1 1 0 0 0 0 0 0 0 0 0 0 0 0 0 1 1 0 0 1 1300 1 37.5 37.5 12.5 12.5

1KC 1 1 0 0 0 0 0 0 1 0 1 1 1 1 1 0 0 0 0 1 0 0 0 0 1 0 1 0 0 0 0 0 0 0 0 0 0 0 0 0 1 1 0 0 1 1340 1 87.5 12.5 12.5 37.5

1KD 1 0 0 0 0 0 0 0 1 1 1 0 1 0 1 0 1 0 1 1 0 1 0 0 1 0 1 0 1 0 0 0 0 0 0 0 0 0 0 0 0 0 0 0 1 1380 1 37.5 37.5 0 0

1KE 1 0 0 0 1 0 0 0 1 1 1 0 1 1 1 0 1 0 1 1 0 1 0 0 1 0 1 0 0 0 0 0 0 0 0 0 0 0 0 0 1 0 0 0 1 1400 1 87.5 62.5 62.5 62.5

1KF 0 1 0 0 0 1 0 0 0 0 0 0 1 1 1 0 1 0 1 0 0 0 1 0 1 0 0 0 0 0 0 0 0 0 0 0 0 0 0 0 0 0 0 0 0 1300 1 12.5 37.5 12.5 6.25

1KG 0 0 1 0 0 0 0 0 1 1 0 0 1 0 0 0 0 0 1 1 0 1 0 0 0 0 1 0 1 0 0 0 0 1 0 0 0 0 0 0 0 0 0 0 1 1500 1 37.5 37.5 0 12.5

1KK 1 0 1 1 0 0 0 0 1 0 0 1 0 0 0 0 0 1 1 1 0 0 0 1 1 0 0 0 0 1 1 0 0 0 0 0 0 0 0 0 0 0 0 0 1 1620 2 62.5 37.5 37.5 62.5

1KL 0 0 1 1 0 0 0 0 1 0 0 1 0 0 0 0 0 1 0 1 0 0 0 0 0 0 0 0 0 0 1 0 0 0 0 0 0 0 0 0 0 0 0 0 0 1770 2 87.5 37.5 37.5 50

1KM 0 0 1 1 0 0 1 0 0 0 0 1 0 0 0 0 0 1 0 0 0 0 0 0 1 0 0 0 0 1 1 0 0 0 0 0 0 0 0 0 0 0 0 0 0 1780 2 87.5 12.5 12.5 12.5

1KN 0 0 1 0 0 1 0 1 1 0 0 0 0 0 0 0 0 1 0 0 0 0 0 0 1 0 0 0 0 1 0 0 0 0 0 0 0 0 0 0 0 0 0 1 0 1840 2 87.5 0 0 25

1KO 0 0 1 1 0 0 0 0 1 0 0 1 0 0 0 0 0 1 0 0 0 0 0 0 1 0 1 0 0 1 0 0 0 1 0 0 0 0 0 0 0 0 0 0 0 1960 2 87.5 37.5 0 62.5

1KP 0 0 0 0 0 0 0 0 0 0 0 0 0 0 0 0 0 0 0 0 0 0 0 0 0 0 1 0 0 0 0 0 0 0 0 0 0 0 0 0 0 0 0 0 0 2010 1 87.5 0 0 0

NA 0 0 1 0 0 1 1 0 0 0 0 1 0 0 0 0 0 1 0 0 0 0 0 1 1 0 0 0 0 1 0 0 0 0 0 0 0 0 0 0 0 0 0 0 0 1660 2 37.5 37.5 87.5 12.5

NA 0 0 1 1 1 1 0 1 0 0 0 0 0 0 0 0 0 1 1 1 0 0 0 0 1 0 0 0 0 1 1 0 0 0 0 0 0 0 0 0 0 0 0 0 0 1800 2 87.5 12.5 12.5 62.5

NA 0 0 1 1 1 0 0 0 0 0 0 1 0 0 0 0 0 1 0 0 0 0 0 0 0 0 0 1 0 0 1 0 0 0 0 0 0 0 1 0 0 0 0 0 0 1900 2 87.5 37.5 37.5 62.5

NA 0 0 1 1 1 0 0 1 0 0 0 0 0 0 0 0 0 1 0 0 0 0 0 0 1 0 0 1 0 0 1 0 0 0 0 0 0 0 0 0 0 0 0 0 0 1960 2 87.5 12.5 62.5 62.5

NA 0 0 1 0 0 0 0 0 0 0 0 1 0 0 0 0 0 1 1 0 0 0 0 0 1 0 1 1 0 1 1 0 0 0 0 0 0 0 1 0 0 0 0 0 0 1980 2 87.5 37.5 37.5 37.5

1LC 0 0 0 0 0 0 0 0 0 1 0 0 0 0 0 0 0 0 1 0 0 0 0 0 0 0 1 0 0 0 0 0 0 0 0 0 0 0 0 0 0 0 0 0 0 1440 1 37.5 37.5 37.5 0

1LD 0 0 0 0 0 0 0 0 0 0 0 1 0 0 1 0 0 0 0 0 0 0 0 0 0 0 1 0 0 0 0 0 0 0 0 0 0 0 0 0 0 0 0 0 0 1500 1 62.5 62.5 37.5 0

1LE 0 0 0 0 0 0 0 0 1 0 0 0 1 0 0 0 0 0 1 0 0 0 0 0 0 0 1 0 0 0 0 1 0 0 0 0 0 0 0 0 0 0 0 0 1 1520 1 37.5 0 0 6.25

1LF 1 0 0 0 0 0 0 0 1 0 0 0 1 1 0 0 1 0 0 0 0 0 1 0 1 0 0 0 0 0 0 0 0 0 0 0 0 0 0 0 0 0 0 0 1 1360 1 0 12.5 37.5 62.5

1LG 1 0 1 0 0 0 0 0 1 0 0 0 0 0 1 0 0 0 1 0 1 0 0 0 1 0 1 0 0 0 0 0 0 0 0 0 0 0 0 0 0 0 0 0 1 1440 1 62.5 37.5 12.5 0

1LH 0 0 1 1 0 0 0 0 1 0 0 0 0 0 1 0 0 0 1 0 0 0 0 1 0 0 1 0 0 0 0 0 0 0 0 0 0 0 0 0 0 0 0 0 1 1580 1 87.5 12.5 37.5 12.5

1LI 0 0 1 1 0 1 0 0 1 0 0 0 0 0 1 1 0 1 1 0 0 0 0 0 0 0 0 0 0 0 0 0 0 0 0 0 0 0 0 0 0 0 0 0 1 1640 2 87.5 62.5 37.5 87.5

1LJ 0 0 1 1 0 0 0 1 0 0 0 1 0 1 0 0 0 1 0 0 0 0 0 0 1 0 0 0 0 1 0 0 0 0 0 0 0 0 0 0 0 0 0 0 0 1730 2 87.5 12.5 62.5 12.5

1LK 0 0 1 1 1 0 0 0 1 0 0 1 0 1 0 0 0 1 1 0 0 0 0 1 1 0 0 0 0 0 1 0 0 0 0 0 0 0 0 0 0 0 0 0 0 1720 2 87.5 87.5 37.5 0

1LM 0 0 1 1 0 1 0 0 1 0 0 1 0 0 0 0 0 1 1 1 0 0 0 0 1 0 0 0 0 1 1 0 0 0 0 0 0 0 0 0 0 0 0 1 0 1800 2 87.5 12.5 12.5 25

1LN 0 0 1 0 0 0 1 1 1 0 0 0 0 0 0 0 0 0 0 0 0 0 0 0 1 0 0 1 0 1 1 0 0 0 0 0 0 0 0 0 0 0 0 0 0 1920 2 87.5 0 0 62.5

1LO 0 0 1 0 0 0 0 0 1 0 0 0 0 0 0 0 0 1 1 0 0 0 0 0 1 0 0 0 0 1 1 0 0 0 0 0 0 0 1 0 0 0 0 0 0 1960 2 87.5 37.5 37.5 0

1LP 0 0 1 1 1 0 0 0 0 0 0 0 0 0 0 0 0 1 0 0 0 0 0 0 1 0 1 0 0 1 1 0 0 0 0 0 0 0 0 0 0 0 0 0 0 2060 2 87.5 37.5 62.5 0

1LQ 0 0 1 0 0 0 0 0 0 0 0 1 0 0 0 0 0 0 1 1 0 0 0 1 1 0 1 0 0 0 0 0 0 0 0 0 0 0 0 0 0 0 0 0 0 2000 1 62.5 62.5 37.5 0

NA 0 0 1 1 1 0 0 0 0 0 0 1 0 0 0 0 0 1 0 0 0 0 0 1 1 0 0 0 0 0 0 0 0 0 0 0 0 0 0 0 0 0 0 0 0 1690 2 87.5 0 0 87.5

NA 0 0 1 0 0 0 0 0 0 0 0 1 0 0 0 0 0 0 0 0 0 0 0 0 1 0 0 0 0 0 1 0 0 0 0 0 0 0 0 0 0 0 0 0 0 1800 2 37.5 37.5 62.5 87.5

NA 0 0 1 0 0 1 1 0 0 0 0 0 0 0 0 0 0 0 0 0 0 0 0 0 1 0 0 0 0 0 0 0 0 0 0 0 0 0 0 0 0 0 0 0 0 1760 2 87.5 37.5 37.5 37.5

NA 0 0 1 1 0 1 1 1 0 0 0 1 0 0 0 0 0 0 0 0 0 0 0 0 1 0 0 0 0 1 0 0 0 0 0 0 0 0 1 0 0 0 0 0 0 1840 2 87.5 37.5 37.5 37.5

NA 0 0 1 1 1 1 0 1 0 0 0 1 0 0 0 0 0 1 1 0 0 0 0 1 1 0 0 0 0 1 1 0 0 0 0 0 0 0 1 0 0 0 0 1 0 1900 2 62.5 62.5 18.75 50

NA 0 0 1 1 0 1 0 1 0 0 0 1 0 0 0 0 0 1 0 0 0 0 0 0 1 0 0 0 0 0 1 0 0 0 0 0 0 0 0 0 0 0 0 1 0 2000 2 87.5 43.75 0 50

1ME 0 0 0 0 0 0 0 0 0 0 0 0 0 0 0 0 0 0 1 0 0 0 0 0 0 1 1 0 0 0 0 0 0 0 0 0 1 0 0 0 0 0 0 0 0 1620 1 37.5 12.5 12.5 0

1MF 0 0 0 0 0 0 0 0 1 0 0 0 1 1 0 0 0 0 0 0 0 0 0 0 1 0 1 0 0 0 0 0 0 0 0 0 0 0 0 0 0 0 0 0 1 1460 1 12.5 37.5 37.5 0

1MG 1 0 0 0 0 1 0 0 1 0 0 0 1 1 0 1 0 0 1 0 0 0 0 0 1 0 0 0 0 0 0 1 1 0 0 0 0 0 0 0 0 0 0 0 0 1360 1 87.5 37.5 12.5 37.5

1MH 0 0 0 0 0 0 0 0 1 0 0 0 1 1 0 1 1 0 1 0 0 0 0 0 0 1 0 0 0 0 0 0 0 0 0 0 0 0 0 0 0 0 0 0 1 1500 1 87.5 37.5 37.5 37.5

1MI 0 0 1 0 0 0 0 0 1 0 0 1 0 0 0 0 0 1 0 0 0 0 0 0 1 0 0 0 0 0 0 0 0 0 0 0 0 0 0 0 1 0 0 0 1 1620 2 87.5 37.5 37.5 62.5

1MJ 0 0 1 1 1 0 0 0 0 0 0 1 0 0 0 0 1 1 1 0 0 0 0 0 0 0 0 0 0 1 1 0 0 0 0 0 0 0 0 0 1 0 0 0 0 1710 2 37.5 37.5 37.5 37.5

1MK 0 0 1 0 1 0 0 1 0 0 0 1 0 0 0 0 0 1 0 1 0 0 0 0 1 0 0 0 0 1 0 0 0 1 0 0 0 0 1 0 0 0 0 0 0 1790 2 87.5 12.5 12.5 62.5

1ML 0 0 1 0 1 1 0 1 1 0 0 1 0 0 0 0 0 0 1 0 0 0 0 0 1 0 0 0 0 0 0 0 0 0 0 0 0 0 0 0 0 0 0 0 0 1880 2 87.5 62.5 37.5 43.75

1MM 0 0 1 1 0 0 0 1 1 0 0 1 0 0 0 0 0 0 0 1 0 0 1 1 1 0 0 1 0 0 0 0 0 0 0 0 0 0 1 0 0 0 0 0 0 1800 2 43.75 0 0 87.5

1MN 0 0 1 0 0 1 0 0 0 0 0 0 0 0 0 0 0 1 0 0 0 0 0 0 1 0 0 0 0 0 0 0 0 0 0 0 0 0 0 0 0 0 0 0 0 1880 2 62.5 0 0 37.5

1MO 0 0 1 1 0 1 0 1 0 0 0 1 0 0 0 0 0 1 0 0 0 0 0 0 1 0 0 0 0 0 1 0 0 0 0 0 0 0 1 0 0 0 0 0 0 1980 2 87.5 0 0 87.5

1MP 0 0 1 0 0 0 0 0 0 0 0 0 0 0 0 0 0 0 1 0 0 0 0 0 1 0 1 0 0 1 0 0 0 0 0 0 0 0 1 0 0 0 0 0 0 2020 2 37.5 31.25 0 43.75

1MQ 0 0 1 0 0 0 0 0 0 0 0 1 0 0 0 0 0 0 1 1 0 0 0 0 1 0 1 1 0 0 0 1 0 0 0 0 0 0 1 0 0 0 0 0 0 2120 1 75 62.5 18.75 0

1MR 0 0 1 0 0 1 0 0 0 0 0 1 0 0 0 0 0 1 0 1 0 0 0 0 1 0 0 0 0 1 0 0 0 0 0 0 0 0 0 0 0 0 0 0 0 1940 1 62.5 87.5 37.5 0

NA 0 0 0 0 0 0 0 0 0 1 0 0 0 0 0 0 0 0 1 0 0 0 0 0 1 0 1 0 0 0 0 1 0 0 0 0 0 0 0 0 0 0 0 0 0 1640 1 37.5 12.5 0 0

NA 0 0 1 1 0 0 0 0 0 0 0 1 0 0 0 0 0 0 1 0 0 0 1 1 0 0 0 0 0 1 1 0 0 0 0 0 0 0 0 0 0 0 0 0 0 1630 2 87.5 25 25 87.5

NA 0 0 1 1 1 0 0 0 0 0 0 1 0 0 0 0 0 1 0 1 0 0 0 0 1 0 0 0 0 1 1 0 0 0 0 0 0 0 0 0 0 0 0 0 0 1740 2 87.5 12.5 12.5 12.5

NA 0 0 1 1 1 0 1 0 0 0 0 1 0 0 0 0 0 1 0 1 0 0 0 0 1 0 0 0 0 0 1 0 0 0 0 0 0 0 0 0 0 0 0 1 0 1820 2 87.5 12.5 12.5 12.5

NA 0 0 1 0 0 0 1 0 0 0 0 1 0 0 0 0 0 1 0 0 0 0 0 0 1 0 0 0 0 0 1 0 0 0 0 0 0 0 0 0 0 0 0 0 0 1900 2 87.5 12.5 12.5 62.5

NA 0 0 1 0 0 1 0 0 0 0 0 1 0 0 0 0 0 1 0 1 0 0 0 1 0 0 0 0 0 0 1 0 0 0 0 0 0 0 1 0 0 0 0 0 0 1800 2 87.5 37.5 37.5 62.5

NA 0 0 1 1 1 0 0 1 0 0 0 1 0 0 0 0 0 1 0 0 0 0 0 0 1 0 0 0 0 0 1 0 0 0 0 0 0 0 0 0 0 0 0 0 0 1940 2 87.5 37.5 12.5 62.5

NA 0 0 1 0 0 0 0 0 1 0 0 1 0 0 0 0 0 0 1 1 0 0 0 0 1 0 1 1 0 1 0 0 0 0 0 0 0 0 1 0 0 0 0 1 0 2030 2 87.5 18.75 18.75 43.75

NA 0 0 0 0 0 0 0 0 0 0 0 0 0 0 0 0 0 0 0 0 0 0 1 0 0 0 1 0 0 0 0 0 0 1 0 0 0 0 0 0 0 0 0 0 0 1700 1 62.5 62.5 12.5 0

1NG 1 0 0 0 0 0 0 0 1 1 0 1 1 1 1 0 0 0 1 0 0 0 0 0 0 0 0 0 0 0 0 0 0 0 0 0 0 0 0 0 0 0 0 0 1 1560 1 50 62.5 31.25 31.25

1NI 0 0 1 0 0 0 0 1 1 0 0 1 1 1 0 1 0 0 1 0 0 0 0 1 1 0 0 0 0 0 0 0 0 0 0 0 0 0 0 0 0 0 0 0 0 1540 2 87.5 37.5 37.5 37.5

1NJ 0 0 1 1 0 1 0 0 0 0 0 0 0 0 0 0 0 0 0 0 0 0 0 1 0 0 0 0 0 0 1 0 0 0 0 0 0 0 0 0 0 0 0 0 1 1640 2 62.5 62.5 62.5 37.5

1NK 0 0 1 1 0 1 0 0 0 0 0 0 0 0 0 0 0 0 1 0 0 0 0 0 1 0 0 0 0 1 0 0 0 0 0 0 0 0 0 0 0 0 0 1 1 1760 2 43.75 62.5 75 6.25

1NL 0 0 1 1 1 0 0 0 0 0 0 1 0 0 0 0 0 1 0 0 0 0 0 0 1 0 0 0 0 1 0 0 0 0 0 0 0 0 1 0 0 0 0 0 0 1850 2 87.5 12.5 12.5 62.5

1NM 0 0 1 1 0 0 0 0 0 0 0 0 0 0 0 0 0 1 0 0 0 0 0 0 1 0 0 0 0 1 1 0 0 0 0 0 0 0 1 0 0 0 0 1 0 1960 2 62.5 12.5 0 62.5

1NN 0 0 1 1 1 0 0 1 0 0 0 0 0 0 0 0 0 1 0 0 0 0 0 1 1 0 0 0 0 1 1 0 0 0 0 0 0 0 1 0 0 0 0 0 1 1960 2 87.5 37.5 37.5 62.5

1NO 0 0 1 0 0 1 0 1 0 0 0 1 0 0 0 0 0 0 0 0 0 0 0 0 1 0 0 0 0 0 1 0 0 0 0 0 0 0 0 0 0 0 0 1 0 2000 2 87.5 12.5 0 62.5

1NP 0 0 0 0 0 0 0 0 0 0 0 0 0 0 0 0 0 0 1 0 0 0 0 0 1 0 1 0 0 1 0 0 1 0 0 0 0 0 0 0 0 0 0 0 0 2100 2 87.5 37.5 37.5 0

1NQ 0 0 1 0 0 0 1 0 0 0 0 1 0 0 0 0 0 0 1 1 0 0 0 1 1 0 1 1 0 1 0 1 0 0 0 0 0 0 0 0 0 0 0 0 0 2110 1 75 0 18.75 0

1NR 0 0 1 0 0 0 0 0 0 0 0 0 0 0 0 0 0 0 0 0 0 0 0 0 1 0 0 0 0 0 0 0 0 0 0 0 0 0 0 0 0 0 0 0 0 2000 1 62.5 62.5 25 0

1NS 0 0 1 0 0 0 0 0 0 0 0 1 0 0 0 0 0 1 0 1 0 0 0 1 1 0 1 0 0 1 0 0 0 0 0 0 0 0 0 0 0 0 0 0 0 2000 1 87.5 62.5 62.5 0

NA 0 0 0 0 0 0 0 0 0 0 0 0 0 0 0 0 0 0 1 0 0 0 0 0 1 0 1 0 0 1 0 0 1 1 0 0 0 0 0 0 0 0 0 0 0 1720 1 12.5 62.5 0 0

NA 0 0 0 0 0 0 0 0 1 0 0 0 1 0 0 0 0 0 0 0 0 0 0 0 0 0 0 0 0 0 0 0 0 0 0 0 0 0 0 0 1 0 0 0 1 1450 1 87.5 31.25 31.25 43.75

NA 0 0 1 0 1 0 0 1 0 0 0 1 0 0 0 0 0 0 1 0 0 0 0 1 1 0 0 0 0 0 0 0 0 0 0 0 0 0 0 0 0 0 0 0 0 1640 2 62.5 37.5 37.5 12.5

NA 0 0 1 1 1 0 0 0 0 0 0 1 0 0 0 0 0 1 1 1 0 0 0 1 1 0 0 0 0 1 1 0 0 0 0 0 0 0 0 0 0 0 0 0 0 1760 2 87.5 37.5 37.5 62.5

NA 0 0 1 1 0 0 0 0 0 0 0 1 0 0 0 0 0 1 0 0 0 0 0 0 1 0 0 0 0 1 1 0 0 0 0 0 0 0 0 0 0 0 0 0 0 1900 2 87.5 0 0 62.5

NA 0 0 1 1 0 0 0 0 0 0 0 1 0 0 0 0 0 1 1 0 0 0 0 0 1 0 0 0 0 1 1 0 0 0 0 0 0 0 1 0 0 0 0 0 0 1980 2 87.5 37.5 37.5 62.5

NA 0 0 1 0 0 0 1 0 0 0 0 1 0 0 0 0 0 1 0 0 0 0 0 0 0 0 0 0 0 1 1 0 0 0 0 0 0 0 0 0 0 0 0 0 0 2080 2 37.5 0 0 50

NA 0 0 1 0 0 0 0 0 0 0 0 1 0 0 0 0 0 1 1 0 0 0 0 0 1 0 0 0 0 1 0 0 0 0 0 0 0 0 0 0 0 0 0 0 0 2080 2 87.5 37.5 37.5 31.25

FX 0 0 0 0 0 0 0 0 1 1 0 0 0 0 0 0 0 0 1 0 0 0 0 0 0 0 0 0 0 0 1 0 0 1 0 0 0 0 0 0 0 0 0 0 1 1700 1 50 62.5 0 0

1OG 0 0 0 0 0 0 0 0 0 0 0 0 0 0 0 0 0 0 1 0 0 0 0 0 0 1 0 0 0 0 0 0 0 0 0 0 0 0 0 0 0 0 0 0 0 1700 1 62.5 37.5 0 0

1OH 0 0 0 0 0 0 0 0 0 0 0 0 0 1 0 0 0 0 1 0 0 0 0 0 0 0 1 0 0 0 0 1 0 0 0 0 1 0 0 0 0 0 0 0 0 1600 1 75 43.75 0 0

1OI 0 0 0 0 0 1 0 0 0 0 0 0 1 1 0 0 0 0 0 0 0 0 0 0 0 0 0 0 0 0 0 0 0 0 0 0 0 0 0 0 0 0 0 0 0 1460 1 37.5 62.5 50 43.75

1OJ 0 0 1 0 0 0 0 1 1 0 0 1 0 0 0 0 0 0 1 0 0 0 0 0 1 0 0 0 0 1 0 0 0 0 0 0 0 0 0 0 0 0 0 0 0 1560 2 87.5 62.5 62.5 43.75

1OK 0 0 1 1 1 1 0 0 0 0 0 1 0 0 0 0 0 1 1 1 0 0 0 1 0 0 0 0 0 1 1 0 0 0 0 0 0 0 0 0 0 0 0 0 0 1700 2 87.5 37.5 50 18.75

1OL 0 0 1 1 1 0 0 0 1 0 0 0 0 0 0 0 0 1 1 1 0 0 0 0 0 0 0 0 0 1 1 0 0 0 0 0 0 0 0 0 0 0 0 0 0 1830 2 62.5 43.75 43.75 62.5

1OM 0 0 1 1 0 0 0 1 0 0 0 1 0 0 0 0 0 1 0 0 0 0 0 0 1 0 0 0 0 1 1 0 0 0 0 0 0 0 1 0 0 0 0 0 0 1930 2 37.5 0 0 62.5

1ON 0 0 1 1 0 0 1 0 0 0 0 0 0 0 0 0 0 0 0 0 0 0 0 0 1 0 0 0 0 1 1 0 0 0 0 0 0 0 1 0 0 0 0 1 0 2060 2 37.5 0 0 62.5

1OO 0 0 1 0 0 0 0 0 1 0 0 0 0 0 0 0 0 0 1 0 0 0 0 0 1 0 1 0 0 1 1 0 1 0 0 0 0 0 0 0 0 0 0 0 0 2140 2 87.5 62.5 62.5 87.5

1OP 0 0 1 0 0 0 0 0 0 0 0 1 0 0 0 0 0 0 1 0 0 0 0 0 1 0 1 0 0 1 0 0 0 1 0 0 0 0 0 0 0 0 0 0 0 2160 1 87.5 18.75 18.75 0

1OR 0 0 1 0 0 0 0 0 0 0 0 0 0 0 0 0 0 0 0 1 0 0 0 0 1 0 1 0 0 0 0 0 0 0 0 0 0 0 0 0 0 0 0 0 0 2140 1 37.5 62.5 37.5 0

NA 0 0 0 0 0 1 0 0 1 0 0 0 0 1 0 0 0 0 1 0 0 0 0 0 1 0 0 0 0 0 0 0 0 0 0 0 0 0 0 0 0 0 0 0 0 1780 1 37.5 87.5 87.5 0

NA 0 0 1 1 0 1 0 1 0 0 0 1 0 0 0 1 0 0 0 0 0 0 1 0 0 0 0 0 0 1 0 0 0 0 0 0 0 0 1 0 0 0 0 0 0 1600 2 87.5 37.5 37.5 87.5

NA 0 0 1 1 1 1 0 0 0 0 0 0 0 0 0 0 0 0 0 1 0 0 0 0 1 0 0 0 0 1 0 0 0 0 0 0 0 0 0 0 0 0 0 0 0 1760 2 87.5 0 0 12.5

NA 0 0 1 1 1 0 1 0 0 0 0 1 0 0 0 0 0 0 1 1 0 0 0 0 1 0 0 0 0 1 1 0 1 1 0 0 0 0 1 0 0 0 0 0 0 1840 2 62.5 0 31.25 62.5

NA 0 0 1 1 1 0 0 0 0 0 0 1 0 0 0 0 0 0 0 0 0 0 0 1 1 0 0 0 0 0 1 0 0 0 0 0 0 0 1 0 0 0 0 0 0 1960 2 75 0 0 62.5

NA 0 0 1 1 0 1 0 1 0 0 0 1 0 0 0 0 0 1 0 0 0 0 0 0 1 0 0 0 0 1 0 0 0 0 0 0 0 0 0 0 0 0 0 0 0 2080 2 62.5 0 0 50

NA 0 0 1 0 0 0 0 1 0 0 0 1 0 0 0 0 0 1 1 0 0 0 0 0 1 0 0 0 0 1 1 0 0 1 0 0 0 0 0 0 0 0 0 0 0 2100 2 87.5 37.5 37.5 37.5

GY 0 0 0 0 0 0 0 0 0 0 0 0 0 0 0 0 0 0 1 0 0 0 0 0 0 1 1 1 0 0 0 0 0 0 0 0 0 0 0 0 0 0 0 0 0 1800 1 62.5 37.5 12.5 12.5

1PH 0 0 0 0 0 0 0 0 0 0 0 0 0 0 0 0 0 0 1 0 0 0 1 0 0 1 1 0 0 0 0 0 0 0 0 0 0 0 0 0 0 0 0 0 0 1920 1 62.5 87.5 0 0

1PI 0 0 0 0 0 0 0 0 0 0 0 0 1 1 0 0 0 0 1 1 0 0 0 0 0 0 0 0 0 0 0 1 0 0 0 0 0 0 0 0 0 0 0 0 0 1620 1 87.5 37.5 12.5 0

1PJ 0 1 0 0 0 1 0 0 1 0 0 1 0 1 0 0 0 0 0 0 0 0 0 0 1 1 0 0 0 0 0 0 0 0 0 0 0 0 0 0 0 0 0 0 0 1500 1 62.5 0 87.5 12.5

1PK 1 0 1 1 0 0 0 0 0 0 0 1 0 0 0 0 1 0 0 0 0 0 0 1 1 0 0 0 0 0 0 0 0 0 0 0 0 0 0 0 0 0 0 0 0 1660 2 87.5 37.5 37.5 62.5

1PL 0 0 1 0 0 1 1 0 0 0 0 0 0 0 0 0 0 0 1 1 0 0 0 0 0 0 0 1 0 1 1 0 1 0 0 0 0 0 1 0 0 0 0 0 0 1800 2 87.5 43.75 43.75 33.33

1PM 0 0 1 0 1 1 0 0 0 0 0 0 0 0 0 0 0 1 0 0 0 0 0 0 1 0 0 0 0 1 1 0 1 0 0 0 0 0 1 0 0 0 0 0 0 1880 2 87.5 0 12.5 62.5

1PN 0 0 1 0 0 1 0 0 0 0 0 1 0 0 0 0 0 0 0 0 0 0 0 0 1 0 0 0 0 0 1 0 0 0 0 0 0 0 0 0 0 0 0 0 0 1980 2 62.5 12.5 12.5 62.5

1PO 0 0 1 0 0 0 0 0 1 0 0 0 0 0 0 0 0 1 0 0 0 0 0 0 1 0 0 0 0 0 1 0 0 0 0 0 0 0 0 0 0 0 0 0 0 2070 2 62.5 62.5 62.5 62.5

1PP 0 0 1 0 0 0 0 1 0 0 0 1 0 0 0 0 0 0 0 0 0 0 0 0 1 0 1 0 0 1 1 0 0 1 0 0 0 0 0 0 0 0 0 0 0 2170 1 87.5 18.75 6.25 31.25

1PQ 0 0 0 0 0 0 0 0 0 0 0 0 0 0 0 0 0 0 0 0 0 0 0 0 0 0 1 0 0 0 0 0 0 0 0 0 0 0 0 0 0 0 0 0 0 2180 1 87.5 0 0 0

NA 0 0 0 0 0 0 0 0 1 1 0 1 0 0 0 0 0 0 1 0 0 1 1 0 1 0 0 1 0 0 0 1 0 0 0 0 0 0 0 0 0 0 0 0 0 1880 1 62.5 62.5 12.5 0

NA 0 0 0 0 0 0 0 0 1 0 0 0 0 1 0 0 0 0 0 0 0 0 0 0 1 1 1 0 0 0 0 0 0 0 0 0 0 0 0 0 0 0 0 0 0 1820 1 87.5 37.5 12.5 0

NA 0 0 1 1 0 1 0 0 0 0 0 1 0 0 0 0 0 0 1 0 0 0 0 1 1 0 0 0 0 0 1 0 1 0 0 0 0 0 0 0 0 0 0 0 0 1800 2 12.5 12.5 37.5 12.5

NA 0 0 1 0 0 1 0 0 0 0 0 1 0 0 0 0 0 1 0 0 0 0 0 0 1 0 0 0 0 0 1 0 0 0 0 0 0 0 1 0 0 0 0 0 0 1880 2 87.5 37.5 12.5 62.5

NA 0 0 1 0 0 0 0 1 0 0 0 1 0 0 0 0 0 1 0 0 0 0 0 1 1 0 0 0 0 0 1 0 0 0 0 0 0 0 0 0 0 0 0 0 0 2000 2 62.5 87.5 0 37.5

NA 0 0 0 0 0 0 1 1 0 0 0 0 0 0 0 0 0 0 0 0 0 0 0 0 0 0 1 0 0 0 1 0 1 0 0 0 0 0 0 0 0 0 0 0 0 2040 2 87.5 12.5 25 37.5

1QF 0 0 0 0 0 1 0 0 1 0 0 0 0 1 0 0 0 0 1 0 0 0 1 0 1 0 1 0 1 0 0 1 0 0 0 0 0 0 0 0 0 0 0 0 0 1860 1 87.5 87.5 12.5 31.25

1QG 0 0 0 0 0 0 1 1 0 0 0 0 0 0 0 0 0 0 1 0 0 0 0 0 0 0 1 0 0 0 0 0 0 0 0 0 0 0 0 0 0 0 0 0 0 1940 1 87.5 43.75 31.25 31.25

1QH 0 0 0 0 0 0 0 0 0 0 0 0 0 0 0 0 0 0 1 0 0 0 0 0 0 0 1 0 0 0 0 1 0 0 0 0 0 0 0 0 0 0 0 0 0 1940 1 37.5 87.5 0 0

1QI 0 0 0 0 0 0 0 0 0 0 0 0 0 1 0 0 0 0 1 0 0 0 0 0 0 0 0 0 1 0 0 1 0 0 0 0 0 0 0 0 0 0 0 0 0 1700 1 75 43.75 0 0

1QJ 1 0 0 0 0 1 0 0 1 1 0 1 0 0 0 0 0 0 1 0 0 0 0 0 1 1 0 0 0 1 0 1 1 0 0 0 0 0 0 0 0 0 0 0 0 1520 1 43.75 62.5 62.5 18.75

1QK 1 0 1 1 0 1 0 1 1 0 0 0 0 1 0 0 0 0 1 0 0 0 0 0 1 1 0 0 0 0 0 0 0 0 0 0 0 0 0 0 0 0 0 0 0 1600 1 62.5 12.5 31.25 0

1QL 0 0 1 1 1 0 0 1 1 0 0 1 0 1 0 1 0 0 1 0 0 0 0 0 0 0 0 0 0 0 0 0 0 0 0 0 0 0 1 0 0 0 0 0 0 1660 2 87.5 12.5 0 87.5

1QM 0 0 1 1 0 1 0 0 0 0 0 1 0 0 0 0 0 1 0 0 0 0 0 0 0 0 0 0 0 0 0 0 0 0 0 0 0 0 0 0 0 0 0 0 0 1800 2 87.5 37.5 0 37.5

1QN 0 0 1 0 0 0 1 0 0 0 0 1 0 0 0 0 0 1 0 0 0 0 0 0 1 0 0 0 0 0 1 0 0 0 0 0 0 0 1 0 0 0 0 0 0 1970 2 87.5 12.5 0 62.5

1QO 0 0 1 0 0 0 0 0 0 0 0 0 0 0 0 0 0 0 0 0 0 0 0 0 1 0 0 0 0 1 1 0 0 0 0 0 0 0 0 0 0 0 0 0 0 2100 2 87.5 12.5 37.5 37.5

1QP 0 0 1 0 0 0 0 1 0 0 0 1 0 0 0 0 0 0 1 0 0 0 0 0 1 0 1 0 0 1 1 0 1 1 0 0 0 0 0 0 0 0 0 0 0 2150 1 87.5 18.75 18.75 0

1QQ 0 0 0 0 0 0 0 0 0 0 0 0 0 0 0 0 0 0 0 0 0 0 0 0 0 0 1 0 0 0 0 0 0 0 0 0 0 0 0 0 0 0 0 0 0 2200 1 87.5 0 0 0

NA 0 0 1 1 1 0 0 0 1 0 0 1 0 0 0 0 0 0 1 1 0 0 0 1 1 0 0 0 0 1 1 0 0 0 0 0 0 0 1 0 0 0 0 0 0 1800 2 87.5 12.5 12.5 18.75

NA 0 0 1 1 1 1 0 0 0 0 0 1 0 0 0 0 0 1 0 0 0 0 0 0 1 0 0 0 0 0 1 0 0 0 0 0 0 0 0 0 0 0 0 0 0 1900 2 62.5 87.5 37.5 62.5

NA 0 0 1 0 0 0 0 1 0 0 0 1 0 0 0 0 0 0 0 0 0 0 0 0 1 0 0 0 0 0 1 0 0 0 0 0 0 0 1 0 0 0 0 0 0 2060 2 12.5 37.5 0 62.5

NA 0 0 0 0 0 0 0 1 0 0 0 0 0 0 0 0 0 0 1 0 0 0 0 0 0 0 1 1 0 1 0 0 1 0 0 0 0 0 0 0 0 0 0 0 0 2100 2 87.5 18.75 18.75 31.25

1RH 0 0 0 0 0 0 0 0 0 0 0 0 0 0 0 0 0 0 1 0 0 0 0 0 0 1 1 0 0 0 0 0 0 0 0 0 0 0 0 0 0 0 0 0 0 2000 1 37.5 12.5 0 0

NA 0 0 0 0 0 0 0 0 0 0 0 0 0 0 0 0 0 0 1 0 0 0 0 0 0 0 0 0 0 0 0 1 0 0 0 0 0 0 0 0 0 0 0 0 0 1700 1 37.5 12.5 12.5 0

1RI 0 0 0 0 0 0 0 0 0 0 0 0 0 0 0 0 0 0 0 0 0 0 0 0 0 0 0 0 1 0 0 1 0 0 0 0 0 0 0 0 0 0 0 0 0 1800 1 37.5 0 0 0

1RJ 0 0 0 0 0 1 0 0 1 0 0 0 1 1 1 0 0 0 0 0 0 0 0 0 0 0 0 0 0 0 0 0 0 0 0 0 0 0 0 0 0 0 0 0 0 1600 1 87.5 37.5 12.5 31.25

1RK 0 0 1 0 0 1 0 0 0 0 0 1 0 1 1 0 0 0 1 0 0 0 0 0 1 1 0 0 0 0 0 0 0 0 0 0 0 0 0 0 0 0 0 0 1 1560 1 62.5 37.5 37.5 31.25

1RL 0 0 1 1 1 0 0 0 1 0 0 0 0 1 0 0 1 1 1 1 0 0 0 0 0 0 0 0 0 0 1 0 0 0 0 0 0 0 0 0 0 0 0 0 0 1700 2 37.5 62.5 62.5 62.5

1RM 0 0 1 1 1 0 0 1 0 0 0 1 0 0 0 0 0 1 0 0 0 0 0 0 0 0 0 0 0 1 0 0 0 0 0 0 0 0 1 0 0 0 0 0 0 1900 2 62.5 37.5 12.5 37.5

1RN 0 0 1 1 0 0 0 0 0 0 0 1 0 0 0 0 0 1 0 0 0 0 0 1 1 0 0 0 0 0 1 0 0 0 0 0 0 0 0 0 0 0 0 0 0 2000 2 62.5 50 0 50

1RO 0 0 1 1 0 0 0 0 0 0 0 0 0 0 0 0 0 1 0 1 0 0 0 0 1 0 0 0 0 1 0 0 0 0 0 0 0 0 0 0 0 0 0 0 0 2120 2 87.5 0 37.5 37.5

1RP 0 0 0 0 0 0 0 0 0 0 0 0 0 0 0 0 0 0 0 0 0 0 0 0 0 0 1 0 0 0 0 0 1 1 0 0 1 0 0 0 0 0 0 0 0 2200 1 87.5 0 0 0

1RQ 0 0 0 0 0 0 0 0 0 0 0 0 0 0 0 0 0 0 0 0 0 0 0 0 0 0 1 0 0 0 0 0 0 0 0 0 0 0 0 0 0 0 0 0 0 2220 1 87.5 0 0 0

NA 0 0 1 1 1 0 0 0 0 0 0 1 0 0 0 1 0 1 0 0 0 0 0 0 1 0 0 0 0 0 0 0 0 0 0 0 0 0 1 0 0 0 0 0 0 1700 2 62.5 12.5 12.5 12.5

NA 0 0 1 1 0 0 0 1 0 0 0 1 0 0 0 1 0 1 1 0 0 0 0 0 0 0 0 0 0 0 1 0 0 0 0 0 0 0 0 0 0 0 0 0 0 1880 2 37.5 62.5 37.5 12.5

NA 0 0 1 1 0 0 0 0 0 0 0 1 0 0 0 0 0 1 1 0 0 0 0 0 1 0 0 1 0 1 1 0 0 0 0 0 0 0 0 0 0 0 0 0 0 2050 2 87.5 12.5 12.5 37.5

NA 0 0 1 0 0 0 0 1 0 0 0 0 0 0 0 0 0 0 0 0 0 0 0 0 0 0 1 0 0 0 1 0 1 0 0 0 0 0 0 0 0 0 0 0 0 2200 2 87.5 50 18.75 0

NA 0 0 0 0 0 0 0 0 0 0 0 0 0 0 0 0 0 0 1 0 0 0 0 0 1 0 1 1 0 1 0 0 1 0 0 0 0 0 0 0 0 0 0 0 0 2200 2 87.5 31.25 31.25 31.25

1SG 0 0 0 0 0 0 0 0 0 0 0 0 0 0 0 0 0 0 1 1 0 0 0 0 0 0 1 1 0 1 0 0 0 0 0 0 0 0 0 0 0 0 0 0 0 2150 1 87.5 18.75 6.25 0

1SH 0 0 0 0 0 0 0 0 0 0 0 0 0 0 0 0 0 0 1 0 0 0 0 0 0 1 0 0 1 0 0 1 0 0 0 0 0 0 0 0 0 0 0 0 0 1950 1 75 31.25 0 0

1SI 0 0 0 0 0 0 0 0 0 0 0 0 0 0 0 0 0 0 1 0 0 0 0 0 0 0 1 0 0 0 0 1 0 0 0 0 1 0 0 0 0 0 0 0 0 1880 1 87.5 62.5 0 0

1SL 0 0 1 1 0 0 0 1 0 0 0 1 0 0 0 0 0 1 0 1 0 0 0 0 1 0 0 0 0 0 0 0 0 0 0 0 0 0 0 0 0 0 0 0 0 1760 2 62.5 62.5 37.5 31.25

1SM 0 0 1 1 0 0 0 0 1 0 0 1 0 0 0 0 0 1 0 0 0 0 0 1 1 0 0 0 0 0 0 0 0 0 0 0 0 0 0 0 0 0 0 0 0 1820 2 87.5 62.5 62.5 50

1SN 0 0 1 1 0 0 0 0 0 0 0 1 0 0 0 0 0 1 0 0 0 0 0 0 0 0 0 0 0 0 1 0 0 0 0 0 0 0 1 0 0 0 0 0 0 1980 2 62.5 0 0 62.5

1SO 0 0 1 1 0 0 0 0 1 0 0 0 0 0 0 0 0 0 0 0 0 0 0 0 1 0 0 0 0 0 1 0 0 0 0 0 0 0 0 0 0 0 0 0 0 2080 2 87.5 37.5 37.5 37.5

1SP 0 0 1 0 0 0 0 1 0 0 0 0 0 0 0 0 0 0 0 0 0 0 0 0 0 0 1 0 0 1 0 0 1 1 0 0 0 0 0 0 0 0 0 0 0 2200 1 75 0 18.75 18.75

1SQ 0 0 0 0 0 0 0 0 0 0 0 0 0 0 0 0 0 0 0 0 0 0 0 0 0 0 1 0 0 0 0 0 0 1 0 0 1 0 0 0 0 0 0 0 0 2200 1 62.5 0 0 0

NA 0 0 1 1 0 0 0 0 0 0 0 0 0 0 0 0 0 1 0 1 0 0 0 1 1 0 0 0 0 0 1 0 0 0 0 0 0 0 0 0 0 0 0 0 0 1850 2 87.5 0 0 87.5

NA 0 0 1 1 0 1 0 0 0 0 0 1 0 0 0 1 0 1 0 0 0 0 0 0 0 0 0 0 0 0 0 0 0 0 0 0 0 0 0 0 0 0 0 0 0 1800 2 62.5 37.5 37.5 37.5

NA 0 0 1 1 0 0 1 0 0 0 0 1 0 0 0 0 0 1 0 0 0 0 0 0 0 0 0 0 0 1 0 0 0 0 0 0 0 0 1 0 0 0 0 0 0 1910 2 87.5 12.5 37.5 37.5

NA 0 0 1 1 1 0 0 0 0 0 0 1 0 0 0 0 0 1 0 0 0 0 0 0 1 0 0 0 0 1 1 0 0 0 0 0 0 0 1 0 0 0 0 0 0 2100 2 62.5 37.5 0 62.5

NA 0 0 1 0 0 0 0 0 0 0 0 1 0 0 0 0 0 0 0 0 0 0 0 0 1 0 1 0 0 1 1 0 1 0 1 0 0 0 0 0 0 0 0 0 0 2140 2 87.5 25 25 62.5

1TH 0 0 0 0 0 0 0 0 0 0 0 0 0 0 0 0 0 0 0 1 0 0 0 0 0 0 1 0 0 0 0 0 0 1 0 0 0 0 0 0 0 0 0 0 0 2150 1 87.5 12.5 12.5 0

1TI 0 0 0 0 0 0 0 0 0 0 0 0 0 0 0 0 0 0 1 0 0 0 0 0 0 1 1 0 1 0 0 1 0 0 0 0 0 0 0 0 0 0 0 0 0 1980 1 62.5 62.5 0 0

1TK 0 0 1 0 1 0 0 0 0 0 0 1 0 0 0 0 0 1 0 0 0 0 0 1 0 0 0 0 0 1 1 0 0 0 0 0 0 0 0 0 0 0 0 0 0 1850 2 0 0 0 87.5

1TL 0 0 1 1 0 0 0 1 0 0 0 1 0 0 0 0 0 0 1 1 0 0 0 0 1 0 0 0 0 1 0 0 0 0 0 0 0 0 0 0 0 0 0 0 0 1930 1 87.5 43.75 18.75 43.75

1TM 0 0 1 1 1 0 1 0 0 0 0 0 0 0 0 0 0 0 0 0 0 0 0 0 1 0 0 0 0 0 0 0 0 0 0 0 0 0 0 0 0 0 0 1 0 1940 2 87.5 62.5 37.5 62.5

1TN 0 0 1 1 0 1 0 0 0 0 0 1 0 0 0 0 0 0 0 0 0 0 0 0 0 0 0 0 0 0 1 0 0 0 0 0 0 0 1 0 0 0 0 0 0 2070 2 62.5 87.5 0 62.5

1TO 0 0 1 1 0 0 1 1 0 0 0 0 0 0 0 0 0 0 1 0 0 0 0 0 1 0 1 0 0 1 1 0 1 0 0 0 0 0 0 0 0 0 0 0 0 2180 2 87.5 18.75 0 18.75

1TP 0 0 0 0 0 0 0 0 0 0 0 0 0 0 0 0 0 0 0 0 0 0 0 0 0 0 1 0 0 0 0 0 0 1 0 0 1 0 0 0 0 0 0 0 0 2250 1 50 6.25 6.25 0

1TQ 0 0 0 0 0 0 0 0 0 0 0 0 0 0 0 0 0 0 0 0 0 0 0 0 0 0 1 0 0 0 0 0 0 1 0 0 1 0 0 0 0 0 0 0 0 2270 1 50 0 0 0

NA 0 0 1 1 1 0 0 1 0 0 0 1 0 0 0 0 0 1 0 0 0 0 0 1 1 0 0 0 0 1 1 0 0 0 0 0 0 0 0 0 0 0 0 0 0 1940 2 87.5 12.5 0 62.5

NA 0 0 1 0 0 0 0 0 0 0 0 1 0 0 0 0 0 1 0 0 0 0 0 0 1 0 0 0 0 1 0 0 0 0 0 0 0 0 0 0 0 0 0 0 0 2100 2 37.5 87.5 0 12.5

NA 0 0 1 0 0 1 0 0 0 0 0 1 0 0 0 0 0 0 0 0 0 0 0 0 0 0 0 1 0 0 1 0 0 0 0 0 0 0 1 0 0 0 0 1 0 2100 2 62.5 37.5 0 62.5

NA 0 0 1 1 1 0 1 1 0 0 0 0 0 0 0 0 0 1 0 0 0 0 0 0 1 0 0 0 0 1 1 0 0 0 0 0 0 0 0 0 0 0 0 0 0 2160 2 37.5 87.5 0 37.5

NA 0 0 0 0 0 0 0 0 0 0 0 0 0 0 0 0 0 0 0 0 0 0 0 0 1 0 1 1 0 0 0 0 0 0 1 0 0 0 0 0 0 0 0 0 0 2260 2 25 43.75 6.25 18.75

1UI 0 0 0 0 0 0 0 0 0 0 0 0 0 0 0 0 0 0 1 1 0 0 0 0 0 0 1 0 0 0 0 1 0 0 0 0 0 0 0 0 0 0 0 0 0 2170 1 62.5 62.5 12.5 0

1UJ 0 0 0 0 0 0 0 0 0 0 0 0 0 0 0 0 0 0 1 1 0 0 0 0 0 0 1 0 1 0 0 1 0 0 0 0 0 0 0 0 0 0 0 0 0 1980 1 62.5 87.5 0 0

1UL 0 0 1 1 0 0 0 0 0 0 0 0 0 0 0 0 0 1 0 0 0 0 0 0 1 0 0 0 0 1 1 0 0 0 0 0 0 0 0 0 0 0 0 0 0 2040 2 87.5 12.5 62.5 62.5

1UM 0 0 1 0 0 0 0 1 1 0 0 0 0 0 0 0 0 0 0 1 0 0 0 0 1 0 1 0 0 1 1 0 0 0 0 0 0 0 0 0 0 0 0 1 0 2130 2 75 18.75 31.25 31.25

1UP 0 0 0 0 0 0 0 0 0 0 0 0 0 0 0 0 0 0 0 0 0 0 0 0 0 0 1 0 0 0 0 0 0 0 0 0 1 0 0 0 0 0 0 0 0 2340 1 50 62.5 0 0

1UQ 0 0 0 0 0 0 0 0 0 0 0 0 0 0 0 0 0 0 0 0 0 0 0 0 0 0 1 0 0 0 0 0 0 0 0 0 1 0 0 0 0 0 0 0 0 2360 1 37.5 37.5 0 0

NA 0 0 1 0 1 1 1 0 0 0 0 1 0 0 0 0 0 1 0 0 0 0 0 0 1 0 0 0 0 1 0 0 0 0 0 0 0 0 0 0 0 0 0 0 0 1940 2 87.5 87.5 0 37.5

NA 0 0 1 1 0 0 0 0 0 0 0 0 0 0 0 0 0 1 1 0 0 0 0 0 1 0 1 1 0 1 1 0 0 0 1 0 0 0 0 0 0 0 0 0 0 2100 2 87.5 12.5 0 6.25

1VJ 0 0 1 0 0 0 0 0 0 0 0 0 0 0 0 0 0 0 1 1 0 0 0 0 0 0 1 0 0 0 0 1 0 0 0 0 0 0 0 0 0 0 0 0 0 2170 1 75 62.5 12.5 0

1VK 0 0 0 0 0 0 0 0 0 0 0 0 0 0 0 0 0 0 1 1 0 0 0 0 0 0 1 0 1 0 0 1 0 0 0 0 0 0 0 0 0 0 0 0 0 2070 1 50 87.5 0 0

1VL 0 0 1 0 0 0 0 0 0 0 0 0 0 0 0 0 0 0 0 0 0 0 0 0 0 0 0 0 0 0 0 0 0 0 0 0 0 0 0 0 0 0 0 0 0 2020 1 12.5 37.5 12.5 0

1VM 0 0 1 0 0 0 1 0 1 0 0 0 0 0 0 0 0 0 1 0 0 0 0 0 1 0 0 1 0 1 0 0 1 0 0 0 0 0 0 0 0 0 0 0 0 2250 1 87.5 12.5 12.5 12.5

1VN 0 0 0 0 0 0 0 0 0 0 0 0 0 0 0 0 0 0 0 0 0 0 0 0 0 0 1 0 0 0 0 0 0 1 0 0 1 0 0 0 0 0 0 0 0 2330 1 37.5 0 0 0

1VO 0 0 0 0 0 0 0 0 0 0 0 0 0 0 0 0 0 0 0 0 0 0 0 0 0 0 1 0 0 0 0 0 0 0 1 0 1 0 0 0 0 0 0 0 0 2370 1 62.5 0 0 0

EZ 0 0 0 0 0 0 0 0 0 0 0 0 0 0 0 0 0 0 0 0 0 0 0 0 0 0 1 0 0 0 0 0 0 0 0 0 0 0 0 0 0 0 0 0 0 2400 1 62.5 0 0 0

1WK 0 0 0 0 0 0 0 0 0 0 0 0 0 0 0 0 0 0 1 0 0 0 0 0 0 0 1 1 0 1 0 0 0 1 0 0 0 0 0 0 0 0 0 0 0 2270 1 87.5 31.25 0 0

1WL 0 0 0 0 0 0 0 0 1 0 0 0 0 0 0 0 0 0 1 0 0 0 0 0 1 0 1 1 0 1 0 0 0 1 0 0 0 0 0 0 0 0 0 0 0 2240 1 75 18.75 0 0

1WN 0 0 0 0 0 0 0 0 0 0 0 0 0 0 0 0 0 0 0 0 0 0 0 0 0 0 1 0 0 0 0 0 0 0 0 0 1 0 0 0 0 0 0 0 0 2440 1 62.5 0 0 0

1WO 0 0 0 0 0 0 0 0 0 0 0 0 0 0 0 0 0 0 0 0 0 0 0 0 0 0 1 0 0 0 0 0 0 0 0 0 1 0 0 0 0 0 0 0 0 2480 1 62.5 0 0 0

1XN 0 0 0 0 0 0 0 0 0 0 0 0 0 0 0 0 0 0 0 0 0 0 0 0 0 0 1 0 0 0 0 0 0 0 1 0 1 0 0 0 0 0 0 0 0 2340 1 37.5 0 0 0

1XO 0 0 0 0 0 0 0 0 0 0 0 0 0 0 0 0 0 0 0 0 0 0 0 0 0 0 1 0 0 0 0 0 0 0 1 0 1 1 0 0 0 0 0 0 0 2460 1 37.5 0 0 0

1YM 0 0 0 0 0 0 0 0 0 0 0 0 0 0 0 0 0 0 0 0 0 0 0 0 0 0 1 0 0 0 0 0 0 0 1 0 1 1 0 0 0 0 0 0 0 2500 1 37.5 0 0 0

1YN 0 0 0 0 0 0 0 0 0 0 0 0 0 0 0 0 0 0 0 0 0 0 0 0 0 0 1 0 0 0 0 0 0 0 1 0 1 0 0 0 0 0 0 0 0 2470 1 37.5 0 0 0

1YO 0 0 0 0 0 0 0 0 0 0 0 0 0 0 0 0 0 0 0 0 0 0 0 0 0 0 1 0 0 0 0 0 1 0 1 0 0 0 0 0 0 0 0 0 0 2450 1 62.5 0 0 0

NA 0 0 0 0 0 0 0 0 0 0 0 0 0 0 0 0 0 0 0 0 0 0 0 0 0 0 1 0 0 0 0 0 0 0 0 0 0 0 0 0 0 0 0 0 0 2530 1 37.5 0 0 0

ZL 0 0 0 0 0 0 0 0 0 0 0 0 0 0 0 0 0 0 0 0 0 0 0 0 0 0 1 0 0 0 0 0 1 0 1 0 1 1 0 0 0 0 0 0 0 2340 1 25 0 0 0

ZM 0 0 0 0 0 0 0 0 0 0 0 0 0 0 0 0 0 0 0 0 0 0 0 0 0 0 1 0 0 0 0 0 1 0 1 0 0 0 0 0 0 0 0 0 0 2600 1 25 0 0 0

ZN 0 0 0 0 0 0 0 0 0 0 0 0 0 0 0 0 0 0 0 0 0 0 0 0 0 0 0 0 0 0 0 0 1 0 1 0 1 1 0 0 0 0 0 0 0 2450 1 50 0 0 0

ZO 0 0 0 0 0 0 0 0 0 0 0 0 0 0 0 0 0 0 0 0 0 0 0 0 0 0 1 0 0 0 0 0 0 0 1 0 0 0 0 0 0 0 0 0 0 2600 1 37.5 0 0 0

ZP 0 0 0 0 0 0 0 0 0 0 0 0 0 0 0 0 0 0 0 0 0 0 0 0 0 0 0 0 0 0 0 0 1 0 1 0 0 1 0 0 0 0 0 0 0 2400 1 37.5 0 0 0

ZQ 0 0 0 0 0 0 0 0 0 0 0 0 0 0 0 0 0 0 0 0 0 0 0 0 0 0 1 0 0 0 0 0 0 0 1 0 1 1 0 0 0 0 0 0 0 2630 1 25 0 0 0

1GC 0 0 0 0 0 0 0 0 0 0 0 0 0 0 0 0 0 0 0 0 1 0 1 0 0 0 1 0 0 0 0 0 0 0 0 1 0 0 0 0 0 0 0 0 0 1215 1 87.5 12.5 12.5 12.5

1SK 0 0 1 0 0 0 0 0 1 1 0 0 0 0 0 0 0 0 1 1 0 0 0 1 0 0 0 0 0 0 0 1 0 0 0 0 0 0 0 0 0 0 0 0 1 1780 1 87.5 37.5 12.5 12.5

1VP 0 0 0 0 0 0 0 0 0 0 0 0 0 0 0 0 0 0 0 0 0 0 0 0 0 0 1 0 0 0 0 0 0 0 0 0 1 0 0 0 0 0 0 0 0 2430 1 62.5 0 0 0
