# Supplementary material for: Combining Niche and Dispersal in a Simple Model (NDM) of Species Distribution
Source: PLoS One. 2013 Nov 12;8(11):e79948. doi: 10.1371/journal.pone.0079948 (PMC3827172; doi:10.1371/journal.pone.0079948)
Supplement: Dataset S3 — Column (Nucol) and line (Nulig) coordinates to map 214 cells out of 302 from dataset S2 on a grid (19 lines and 24 columns). Code is to do the correspondence with dataset S2. (DOC) [file pone.0079948.s005.doc]

**Dataset S3**. Column (Nucol) and line (Nulig) coordinates to map 214 cells out of 302 from dataset S2 on a grid (19 lines and 24 columns). Code is to do the correspondence with dataset S2.

Nucol Nulig Code

1 1 1EA

1 2 1EB

1 3 1EC

1 4 1ED

1 5 1EE

1 6 1EF

2 1 1FA

2 2 1FB

2 3 1FC

2 5 1FE

2 6 1FF

2 7 1FG

2 8 1FH

2 9 1FI

2 10 1FJ

2 11 1FK

3 1 1GA

3 2 1GB

3 5 1GE

3 6 1GF

3 7 1GG

3 8 1GH

3 9 1GI

3 10 1GJ

3 11 1GK

3 12 1GL

3 13 1GM

4 1 1HA

4 2 1HB

4 5 1HE

4 6 1HF

4 7 1HG

4 8 1HH

4 9 1HI

4 10 1HJ

4 11 1HK

4 12 1HL

4 13 1HM

5 1 1IA

5 2 1IB

5 3 1IC

5 5 1IE

5 6 1IF

5 7 1IG

5 8 1IH

5 9 1II

5 10 1IJ

5 11 1IK

5 12 1IL

5 13 1IM

5 14 1IN

5 15 1IO

6 1 1JA

6 2 1JB

6 3 1JC

6 4 1JD

6 5 1JE

6 6 1JF

6 7 1JG

6 8 1JH

6 9 1JI

6 10 1JJ

6 11 1JK

6 12 1JL

6 13 1JM

6 14 1JN

6 15 1JO

6 16 1JP

7 1 1KA

7 3 1KC

7 4 1KD

7 5 1KE

7 6 1KF

7 7 1KG

7 11 1KK

7 12 1KL

7 13 1KM

7 14 1KN

7 15 1KO

7 16 1KP

8 3 1LC

8 4 1LD

8 5 1LE

8 6 1LF

8 7 1LG

8 8 1LH

8 9 1LI

8 10 1LJ

8 11 1LK

8 13 1LM

8 14 1LN

8 15 1LO

8 16 1LP

8 17 1LQ

9 5 1ME

9 6 1MF

9 7 1MG

9 8 1MH

9 9 1MI

9 10 1MJ

9 11 1MK

9 12 1ML

9 13 1MM

9 14 1MN

9 15 1MO

9 16 1MP

9 17 1MQ

9 18 1MR

10 7 1NG

10 9 1NI

10 10 1NJ

10 11 1NK

10 12 1NL

10 13 1NM

10 14 1NN

10 15 1NO

10 16 1NP

10 17 1NQ

10 18 1NR

10 19 1NS

11 5 FX

11 7 1OG

11 8 1OH

11 9 1OI

11 10 1OJ

11 11 1OK

11 12 1OL

11 13 1OM

11 14 1ON

11 15 1OO

11 16 1OP

11 18 1OR

12 7 GY

12 8 1PH

12 9 1PI

12 10 1PJ

12 11 1PK

12 12 1PL

12 13 1PM

12 14 1PN

12 15 1PO

12 16 1PP

12 17 1PQ

13 6 1QF

13 7 1QG

13 8 1QH

13 9 1QI

13 10 1QJ

13 11 1QK

13 12 1QL

13 13 1QM

13 14 1QN

13 15 1QO

13 16 1QP

13 17 1QQ

14 8 1RH

14 9 1RI

14 10 1RJ

14 11 1RK

14 12 1RL

14 13 1RM

14 14 1RN

14 15 1RO

14 16 1RP

14 17 1RQ

15 7 1SG

15 8 1SH

15 9 1SI

15 12 1SL

15 13 1SM

15 14 1SN

15 15 1SO

15 16 1SP

15 17 1SQ

16 8 1TH

16 9 1TI

16 11 1TK

16 12 1TL

16 13 1TM

16 14 1TN

16 15 1TO

16 16 1TP

16 17 1TQ

17 9 1UI

17 10 1UJ

17 12 1UL

17 13 1UM

17 16 1UP

17 17 1UQ

18 10 1VJ

18 11 1VK

18 12 1VL

18 13 1VM

18 14 1VN

18 15 1VO

18 16 EZ

19 11 1WK

19 12 1WL

19 14 1WN

19 15 1WO

20 14 1XN

20 15 1XO

21 13 1YM

21 14 1YN

21 15 1YO

22 12 ZL

22 13 ZM

23 12 ZN

23 13 ZO

24 12 ZP

24 13 ZQ

3 3 1GC

15 11 1SK

16 18 1VP
